# PBTK modeling of the pyrrolizidine alkaloid retrorsine to predict liver toxicity in mouse and rat

**DOI:** 10.1007/s00204-023-03453-z

**Published:** 2023-03-11

**Authors:** Anja Lehmann, Ina Geburek, Anja These, Stefanie Hessel-Pras, Jan G. Hengstler, Wiebke Albrecht, Hans Mielke, Christine Müller-Graf, Xiaojing Yang, Charlotte Kloft, Christoph Hethey

**Affiliations:** 1 German Federal Institute for Risk Assessment (BfR), Max-Dohrn-Str. 8-10, 10589 Berlin, Germany; 2 Department of Clinical Pharmacy and Biochemistry, Institute of Pharmacy, Freie Universitaet Berlin, 12169 Berlin, Germany; 3 Leibniz Research Centre for Working Environment and Human Factors (IfADo), Technical University of Dortmund, 44139 Dortmund, Germany; 4 Wuya College of Innovation, Shenyang Pharmaceutical University, Shenyang, 110016 Liaoning People’s Republic of China

**Keywords:** PBTK, Toxicokinetics, Retrorsine, Pyrrolizidine alkaloids, Hepatotoxicity, Benchmark dose analysis

## Abstract

Retrorsine is a hepatotoxic pyrrolizidine alkaloid (PA) found in herbal supplements and medicines, food and livestock feed. Dose-response studies enabling the derivation of a point of departure including a benchmark dose for risk assessment of retrorsine in humans and animals are not available. Addressing this need, a physiologically based toxicokinetic (PBTK) model of retrorsine was developed for mouse and rat. Comprehensive characterization of retrorsine toxicokinetics revealed: both the fraction absorbed from the intestine (78%) and the fraction unbound in plasma (60%) are high, hepatic membrane permeation is dominated by active uptake and not by passive diffusion, liver metabolic clearance is 4-fold higher in rat compared to mouse and renal excretion contributes to 20% of the total clearance. The PBTK model was calibrated with kinetic data from available mouse and rat studies using maximum likelihood estimation. PBTK model evaluation showed convincing goodness-of-fit for hepatic retrorsine and retrorsine-derived DNA adducts. Furthermore, the developed model allowed to translate in vitro liver toxicity data of retrorsine to in vivo dose-response data. Resulting benchmark dose confidence intervals (mg/kg bodyweight) are 24.1–88.5 in mice and 79.9–104 in rats for acute liver toxicity after oral retrorsine intake. As the PBTK model was built to enable extrapolation to different species and other PA congeners, this integrative framework constitutes a flexible tool to address gaps in the risk assessment of PA.

**Supplementary Information:**

The online version contains supplementary material available at 10.1007/s00204-023-03453-z.

## Introduction

Retrorsine is one of the most frequently occurring pyrrolizidine alkaloids in herbal teas along with senecionine and seneciphylline (Mulder et al. 2018; Schulz et al. 2015). The compound is a member of 1,2-unsaturated pyrrolizidine alkaloids (PA), a structurally diverse group of secondary metabolites, produced in a wide variety of plants (Roeder 1995). PA are found in herbal teas, in food such as honey and spices, in herbal food supplements and medicines (e.g. St. John’s Wort), as well as in livestock feed.

Due to their undesired hepatotoxic, genotoxic and carcinogenic properties, PA are subject to a comprehensive risk assessment (BfR 2020; Chen et al. 2017; Dusemund et al. 2018; EFSA 2017a; EMA 2021). Hepatic DNA adducts, as well as primary liver tumors were observed in rats after oral administration of retrorsine (Schoental et al. 1954; Wang et al. 2005). Dose-response studies allowing the derivation of a benchmark dose for liver toxicity of retrorsine are not available in both humans and animals. Previously, the European Food Safety Authority has emphasized that information on toxicokinetics, metabolic activation and carcinogenic potency of individual PA is currently incomplete and generation of such data would greatly contribute to the refinement of risk assessment (EFSA 2017a).

PA are protoxins that become metabolically activated in the liver via oxidation by cytochrome P450 (CYP) enzymes. Upon oxidation, reactive dehydro-PA metabolites are formed that bind to DNA, proteins and glutathione (GSH). DNA adducts and protein adducts are emerging biomarkers for carcinogenicity and hepatotoxicity of PA (He et al. 2017; Ma et al. 2018). GSH conjugates are considered detoxification metabolites, however, they also have been reported to form toxic DNA adducts after hydrolysis in vitro (Xia et al. 2015).

In addition to hepatic metabolism, hepatic transport has recently been recognized as a key determinant of PA toxicity. The role of two influx transporters, the organic cation transporter 1 (OCT1) and the sodium/taurocholate co-transporting polypeptide (NTCP), in the hepatic uptake of retrorsine was demonstrated in vitro using cultivated primary rat hepatocytes, hOCT1-expressing Madin–Darby canine kidney cells and/or the human hepatoma cell line HepaRG (Enge et al. 2021; Tu et al. 2014).

The potency of PA toxicity is highly dependent on the chemical structure of PA congeners and marked species differences in susceptibility to PA toxicity exist (He et al. 2021; Merz and Schrenk 2016). For a profound understanding of congener- and species-specific differences in PA toxicity an understanding of the underlying PA toxicokinetics is crucial.

Physiologically-based toxicokinetic (PBTK) models have demonstrated their capability to mechanistically understand the interplay of hepatic metabolism and transport (Hanke et al. 2018). By linking defined external exposure scenarios to internal tissue concentrations, PBTK models allow a functional understanding of absorption, tissue distribution and elimination of compounds. The physiology-based method facilitates the translation of toxicokinetics between species (Thiel et al. 2015). It further enables the extrapolation of administration routes often used in animal studies, such as intravenous and intraperitoneal administration, to oral uptake, which is relevant for human risk evaluation (Lin and Wong 2017).

The benefit of PBTK modeling for the prediction of in vivo acute liver toxicity and/or in vivo genotoxicity based on in vitro toxicity data has been demonstrated and evaluated for other PA lasiocarpine, riddelliine and monocrotaline (Chen et al. 2018, 2019; Suparmi et al. 2020).

The first objective of this article was to develop a PBTK model of retrorsine with a focus on liver metabolism and transport. For this purpose, we comprehensively characterized retrorsine toxicokinetics using in silico models and in vitro experiments. By means of in vitro-to-in vivo extrapolation, we informed key model parameters to understand the interplay of retrorsine hepatic transport and metabolism. The second objective was to evaluate the PBTK model. Development and evaluation of the PBTK model rely on the availability of in vivo kinetic data. Retrorsine is one of the few PA for which in vivo mouse or rat kinetic data has been reported. Finally, we used the evaluated PBTK model for the prediction of acute liver toxicity of retrorsine in vivo based on toxicity data in primary mouse and rat hepatocytes. In this approach, we infered organ toxicity from in vitro cytotoxicity. Therefore, PBTK model-based simulation of retrorsine liver concentrations was performed to identify the corresponding retrorsine doses provoking the measured toxic response (reverse dosimetry). Predicted in vivo dose-response data were used to derive benchmark dose confidence intervals for acute liver toxicity in mouse and rat.

## Methods

### Collection of kinetic data from animal studies

Kinetic in vivo studies with retrorsine in mouse and rat were identified by a PubMed search (www.ncbi.nlm.nih.gov) using the term ’retrorsine AND (kinetics OR excretion OR bioavailability)’. Exclusion criteria were (i) in vitro studies, (ii) in vivo studies not reporting measurements of retrorsine or its metabolites and (iii) duplicate studies.

Most studies reported observations in form of summary data (mean, standard deviation, number of observations) instead of individual data. To use summary data jointly with individual data, summary data were ’de-aggregated’ as outlined in Hethey et al. (2021). Kinetic data from independently performed studies were reported with different units. To be able to use kinetic data jointly for model development, they were standardized into the common base unit ’amount of substance’ (nmol). Detailed protocols of this standardization are provided as Supplementary Information ’Pre-processing of in vivo kinetic data’ (Eqs. S16–S22). Graphical data were extracted using the online tool WebPlotDigitizer (Rohatgi 2020).

### PBTK model structure

**Fig. 1:**
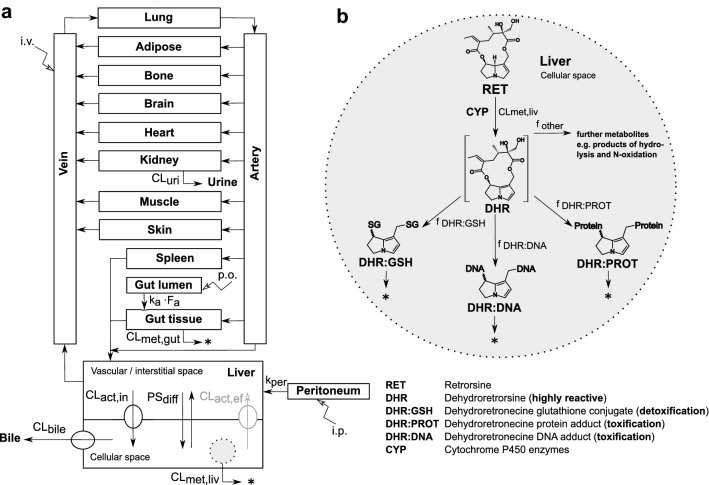
Structure of the PBTK model of retrorsine (**a**). The liver compartment was represented by the extended clearance model accounting for transport and metabolism. Retrorsine was administered as intraperitoneal (i.p.), intravenous (i.v.) or per oral (p.o.) solution. Simplified model of retrorsine metabolism in the liver cellular space **b** including metabolites DHR:GSH, DHR:PROT and DHR:DNA, for which in vivo data were available from kinetic studies. All model parameters are summarized in Table S1. The full system of ordinary differential equations is given in Eqs. S1–S15. $\textrm{CL}_{\rm{act},\text{in}}$ active uptake clearance, $\textrm{CL}_{\rm{act},\text{ef}}$ active efflux clearance, $\textrm{CL}_{\textrm{bile}}$ biliary clearance, $\textrm{CL}_{\rm{met},\text{gut}}$ gut metabolic clearance, $\textrm{CL}_{\rm{met},\text{liv}}$ liver metabolic clearance, $\textrm{CL}_{\textrm{uri}}$ renal clearance, $F_{\textrm{a}}$ intestinal fraction absorbed, $f_{\textrm{DHR}:\textrm{DNA}}$ fraction metabolized to DHR:DNA, $f_{\textrm{DHR}:\textrm{GSH}}$ fraction metabolized to DHR:GSH, $f_{\textrm{DHR}:\textrm{PROT}}$ fraction metabolized to DHR:PROT, $f_{\textrm{other}}$ fraction metabolized to other retrorsine metabolites, $k_{\textrm{a}}$ intestinal absorption rate constant, $k_{\textrm{per}}$ peritoneal absorption rate constant, $\textrm{PS}_{\textrm{diff}}$ passive influx diffusion flow rate

The PBTK model of retrorsine is based on a generic whole-body structure with 13 anatomical compartments (Fig. 1a) (Hartung and Huisinga 2019; Pilari and Huisinga 2010). As retrorsine is a known substrate of the sinusoidal influx transporters OCT1 and NTCP (Enge et al. 2021; Tu et al. 2014), the liver compartment of the generic PBTK model was adapted according to the extended clearance model. This organ model integrates active and passive transport next to metabolism into the liver compartment (Patilea-Vrana and Unadkat 2016; Schweinoch 2014; Sirianni and Pang 1997).

The liver compartment was separated into a lumped vascular/interstitial (superscript vi) space and a cellular (superscript c) space. Equation 1 illustrates, how the amount of retrorsine in the liver cellular space $\textrm{RET}_{\rm{liv}}^{c}(t)$ changes over time *t* as a result of an interplay of hepatic processes:

1 $$\begin{aligned} \frac{\text{d}\rm{RET}_{\text{liv}}^{c} (t)}{\rm{d}t} =&\,\, \text {Active and passive influx} - \rm {Active and passive efflux}\nonumber \\&- \text {Biliary excretion} - \rm {Metabolic elimination} \end{aligned}$$

where:

- $${\text{Active and passive influx }} = \left( {{\rm{CL}}_{{{\text{act}},{\rm{in}}}} + {\text{PS}}_{{{\rm{diff}}}} } \right) \cdot K_{{{\text{liv}}}}^{{{\rm{int}},{\text{u}}:{\rm{vi}}}} \cdot \frac{{{\text{RET}}_{{{\rm{liv}}}}^{{{\text{vi}}}} (t)}}{{V_{{{\rm{liv}}}}^{{{\text{vi}}}} }},$$
- $${\text{Active and passive efflux }} = \;\left( {{\text{CL}}_{{{\text{act}},{\text{ef}}}} {\text{ + PS}}_{{{\text{diff}}}} \cdot \frac{{{\text{fn}}_{{{\text{liv}}}}^{{\text{c}}} }}{{{\text{fn}}_{{{\text{liv}}}}^{{{\text{int}}}} }}} \right) \cdot {\text{fu}}_{{{\text{liv}}}}^{{\text{c}}} \cdot \frac{{{\text{RET}}_{{{\text{liv}}}}^{{\text{c}}} ({\text{t}})}}{{{\text{V}}_{{{\text{liv}}}}^{{\text{c}}} }},$$
- $${\text{Biliary excretion }} = {\text{CL}}_{{{\rm{bile}}}} \cdot {\text{fu}}_{{{\rm{liv}}}}^{c} \cdot \frac{{{\text{RET}}_{{{\rm{liv}}}}^{c} (t)}}{{V_{{{\text{liv}}}}^{c} }},$$
- $${\text{Metabolic elimination }} = \quad 1 \cdot \frac{{V_{{{\text{max}},{\rm{liv}}}} }}{{K_{{{\text{M}},{\rm{liv}}}} + {\text{fu}}_{{{\rm{liv}}}}^{c} \cdot \frac{{{\text{RET}}_{{{\rm{liv}}}}^{{\text{c}}} (t)}}{{V_{{{\rm{liv}}}}^{c} }}}} \cdot {\text{fu}}_{{{\rm{liv}}}}^{c} \cdot \frac{{{\text{RET}}_{{{\rm{liv}}}}^{c} (t)}}{{V_{{{\text{liv}}}}^{c} }},\quad {\rm{with }}{\mkern 1mu} 1 = f_{{{\text{DHR}}:{\rm{GSH}}}} + f_{{{\text{DHR}}:{\rm{DNA}}}} + f_{{{\text{DHR}}:{\rm{PROT}}}} + f_{{{\text{other}}}}. $$

Retrorsine is transported into and out of the liver cellular space via active carrier-mediated transport ($\textrm{CL}_{\rm{act},\text{in}}$ active influx clearance; $\text{CL}_{\rm{act},\text{ef}}$ active efflux clearance) and via passive diffusion ($\text{PS}_{\rm{diff}}$ passive influx diffusion flow rate; $\textrm{PS}_{\textrm{diff}}\cdot \textrm{fn}_{\textrm{liv}}^{c}/\textrm{fn}_{\textrm{liv}}^{\textrm{int}}$ passive efflux diffusion flow rate; $\textrm{fn}_{\textrm{liv}}^{c}$ and $\textrm{fn}_{\textrm{liv}}^{\textrm{int}}$ representing the fraction neutral in the liver cellular and interstitial space, respectively). From the liver cellular space retrorsine is either excreted into the bile ($\textrm{CL}_{\textrm{bile}}$ biliary clearance) or eliminated via metabolism ($K_{\textrm{M},\textrm{liv}}$ Michaelis-Menten constant; $V_{\textrm{max},\textrm{liv}}$ maximum reaction velocity). In Eq. 1 the term $K_{\textrm{liv}}^{\textrm{int},\textrm{u}:\textrm{vi}} \cdot \textrm{RET}_{\textrm{liv}}^{c} (t)/V_{\textrm{liv}}^{\textrm{c}}$ represents the unbound retrorsine concentration in the lumped vascular/interstitial space and the term $\textrm{fu}_{\textrm{liv}}^{c}\cdot \textrm{RET}_{\textrm{liv}}^{\textrm{c}} (t)/V_{\textrm{liv}}^{c}$ represents the unbound retrorsine concentration in the liver cellular space ($K_{\textrm{liv}}^{\textrm{int},\textrm{u}:\textrm{vi}}$ liver unbound interstitial-to-lumped compartment partition coefficient; $\textrm{fu}_{\textrm{liv}}^{c}$ fraction unbound in the liver cellular space; $V_{\textrm{liv}}^{c}$ volume of liver cellular space).

A simplified model of metabolism was added to the liver cellular space (Fig. 1b). Retrosine (RET) is oxidized by CYP enzymes to dehydroretrorsine (DHR), which is a key step in retrorsine toxicity. Isoforms mainly involved in the oxidation of retrorsine were CYP2A and CYP3A as shown with human recombinant CYP supersomes (Ruan et al. 2014). DHR is a chemically reactive and electrophilic intermediate and was not included in the model. The short timescale of subsequent reactions to form stable products impairs the direct quantification in vivo and in vitro. Indirect quantification of DHR via measurement of stable end-products including DNA adducts (DHR:DNA), protein adducts (DHR:PROT) and glutathione conjugates (DHR:GSH) is commonly performed and was included in the model as a surrogate. The fractional formation of DNA adducts, protein adducts and glutathione conjugates was modeled as a first-order process. Further major retrorsine metabolites like retrorsine N-oxide or products of retrorsine hydrolysis, for which no kinetic data were available from animal studies, were considered as a fraction of other metabolites ($f_{\textrm{other}}$). Depletion of hepatic DNA adducts was described by a biexponential model ($\lambda _{1,\textrm{DHR}:\textrm{DNA}}$ 1st phase depletion rate constant; $\lambda _{2,\textrm{DHR}:\textrm{DNA}}$ 2nd phase depletion rate constant; $k_{\textrm{DHR}:\textrm{DNA}}$ transition rate constant), while depletion of hepatic protein adducts and GSH conjugates was described by a monoexponential model ($\lambda _{\textrm{DHR}:\textrm{PROT}}$; $\lambda _{\textrm{DHR}:\textrm{GSH}}$ depletion rate constant). The full system of ordinary differential equations is given in Eqs. S1–S15. All model parameters are summarized in Table S1.

### Parametrization of absorption, tissue distribution and elimination

Intraperitoneal injection was modeled as first-order absorption from a peritoneum compartment (Eq. S1). Peritoneal absorption was assumed to be fast with a half-life of 15 sec (corresponding to an absorption rate constant $k_{\textrm{per}}$ of 166 1/h).

Oral absorption was modeled as a first-order process from the gut lumen compartment into the gut tissue (Eq. S2). The intestinal absorption rate constant $k_{\textrm{a}}$ was estimated based on available kinetic data from a mouse oral study (Li et al. 2022). The fraction absorbed from the gut lumen $F_{\textrm{a}}$ was predicted based on a reported correlation between in vitro Caco-2 permeability and $F_{\textrm{a}}$ [Eq. S35, Skolnik et al. (2010)]. Therefore, in vitro permeability assays were performed with a Caco-2 monolayer in a Transwell system as a model for the intestinal barrier (protocol as Supplementary Information ’Caco-2 Permeability Assay’).

Tissue-to-plasma partition coefficients $K_{\textrm{tis}}$ are needed to describe the steady-state concentration of retrorsine in the tissues compared to blood plasma. $K_{\textrm{tis}}$ of retrorsine were predicted for perfusion-limited anatomical compartments using an established tissue distribution model (Rodgers and Rowland 2006). The mechanistic model approximates distribution into tissues by assuming distribution into the main tissue constituents (water, neutral lipids, phospholipids, proteins) instead. The extend of tissue distribution depends on the physico-chemical properties of retrorsine. Our prediction of $K_{\textrm{tis}}$ was generated based on a MATLAB pharmacometric modeling framework developed by Hartung and Huisinga (2019).

For the lumped vascular/interstitial liver compartment, partition coefficients $K_{\textrm{liv}}^{\textrm{vas}:\textrm{vi}}$ and $K_{\textrm{liv}}^{\textrm{int},\textrm{u}: \textrm{vi}}$ were used, which consider the concentration differences between vascular and interstitital space (Eq. S10; derived by Schweinoch (2014)).

To parametrize the extended clearance model of the liver, two in vitro experiments were performed: the liver microsomal assay and the medium loss assay. The Michaelis-Menten constant $K_{\textrm{M},\textrm{liv}}$ and the maximum reaction velocity $V_{\textrm{max},\textrm{liv}}$ of hepatic metabolism were predicted from mouse and rat liver microsomal assays (Eqs. S28–S32). Medium loss assays with mouse and rat primary hepatocytes were performed under physiological (37 °C) and non-physiological (4 °C), non-saturating conditions to predict active uptake clearance $\textrm{CL}_{\textrm{act},\textrm{in}}$ and passive influx diffusion flow rate $\textrm{PS}_{\textrm{diff}}$ (Eqs. S24–S27, Fig. S1). Experimental protocols are provided as Supplementary Information (’Liver microsomal assay’, ’Isolation and Culture of Primary Hepatocytes’, ’Medium Loss Assay’). Biliary clearance $\textrm{CL}_{\textrm{bile}}$ was estimated from retrorsine amounts in rat bile (White 1977). Active efflux clearance $\textrm{CL}_{\textrm{act},\textrm{ef}}$ was assumed negligible. Overall liver clearance $\textrm{CL}_{\textrm{liv}}$, also affected by hepatic blood flow $Q_{\textrm{liv}}$ and the retrorsine fraction unbound in blood plasma (fuP), was calculated according to Eq. 2 (Patilea-Vrana and Unadkat 2016).

2 $$\begin{aligned} \textrm{CL}_{\textrm{liv}}=&\, \frac{ Q_{\textrm{liv}} \cdot \textrm{fuP}\cdot \textrm{CL}_{\textrm{act},\textrm{in}} \cdot \left( \textrm{CL}_{\textrm{met},\textrm{liv}} + \textrm{CL}_{\textrm{bile}}\right) }{Q_{\textrm{liv}} \cdot \left( \textrm{CL}_{\textrm{act},\textrm{ef}} + \textrm{CL}_{\textrm{met},\textrm{liv}} + \textrm{CL}_{\textrm{bile}}\right) + \textrm{fuP}\cdot \textrm{CL}_{\textrm{act},\textrm{in}} \cdot \left( \textrm{CL}_{\textrm{met},\textrm{liv}} + \textrm{CL}_{\textrm{bile}}\right) }\nonumber \\&\text {with } \textrm{CL}_{\textrm{met},\textrm{liv}} = \frac{V_{\textrm{max},\textrm{liv}}}{K_{\textrm{M},\textrm{liv}}}. \end{aligned}$$

Extrahepatic elimination routes included in the PBTK model were renal excretion and intestinal metabolism. With regard to intestinal metabolism, it was assumed that the Michaelis-Menten constant $K_{\textrm{M},\textrm{gut}}$ is identical to that of hepatic metabolism and the maximum reaction velocity $V_{\textrm{max},\textrm{gut}}$ is reduced by factor 10 compared to hepatic metabolism. This assumption was based on a comparative analysis of the hepatic and intestinal metabolic activity of CYP3A substrates in human microsomes by Galetin and Houston (2006). The authors observed $K_{\textrm{M},\textrm{gut}}$ values within 2-fold of hepatic estimates and $V_{\textrm{max},\textrm{gut}}$ values reduced by factor 4.5–50 compared to hepatic estimates.

### Parameter estimation and model evaluation

Unknown parameters of the PBTK model were estimated based on *in vivo* data from animal studies. These parameters include the intestinal absorption rate constant $k_{a}$, the rat biliary clearance CL$_{\textrm{bile}}$ and parameters related to formation and depletion of hepatic metabolites DHR:GSH, DHR:DNA and DHR:PROT, i.e. fractions metabolized $f_{\textrm{DHR}:\textrm{GSH}}$, $f_{\textrm{DHR}:\textrm{PROT}}$, $f_{\textrm{DHR}:\textrm{DNA}}$, (1st phase) depletion rate constants $\lambda _{\textrm{DHR}:\textrm{GSH}}$, $\lambda _{\textrm{DHR}:\textrm{PROT}}$, $\lambda _{1,\textrm{DHR}:\textrm{DNA}}$, 2nd phase depletion rate constant $\lambda _{2,\textrm{DHR}:\textrm{DNA}}$ and transition rate constant $k_{\textrm{DHR}:\textrm{DNA}}$.

Parameter estimation was performed via maximum likelihood estimation using Monte Carlo Markov Chain (MCMC) analysis. The objective function value -2$\cdot$ln(likelihood) was used as a numeric criterion to assess the quality of model fit. MCMC generates a posterior distribution of the optimized parameter vector by iteratively drawing samples from a probability distribution based on the likelihood. The optimization algorithm implemented in the MCMC analysis was Metropolis-Hastings using the Delayed Rejection and Adaptive Metropolis procedure [R package FME, Haario et al. (2006), Soetaert and Petzoldt (2010)]. Three MCMC chains with non-informative prior and overdispersed initial values were generated and convergence was assumed, if the Gelman and Rubin’s convergence diagnostic was smaller than 1.1 [R package coda, Plummer et al. (2006)]. Parameter estimates were reported as mode and [95% credible interval] of the posterior distribution [R package bayestestR, Makowski (2019)].

The predictive performance of the PBTK model was evaluated based on independent in vivo evaluation data from mouse studies (Table S2 column ’Function’). Parameter uncertainty was assessed numerically by [95% credible interval] and graphically by pairs plots of 1000 MCMC samples.

All modeling activities were performed using the software R Version 4.0.5 (RCoreTeam 2021) and RStudio Version 1.4.1106 (RStudioTeam 2021). Further R packages used were dfoptim providing derivative-free optimization algorithms (Varadhan et al. 2020), RxODE for solving ordinary differential equation systems and for model-based simulations (Wang et al. 2016) and ggplot2 for the generation of figures (Wickham 2016). All figures were finalized using Inkscape (InkscapeProject 2020).

### Reverse dosimetry and benchmark dose analysis

The PBTK model was applied to predict hepatotoxic dose levels after single oral retrorsine administration in mouse and rat based on in vitro liver toxicity data. Primary mouse and rat hepatocytes were used as in vitro model for acute liver toxicity. Isolated hepatocytes were cultured for 3 h after seeding followed by cytotoxicity testing with retrorsine for an additional 48 h (experimental protocol provided as Supplementary Information ’Isolation and Culture of Primary Hepatocytes’, ’Cytotoxicity Assay’). Concentration-response data generated from this in vitro model were translated into in vivo dose-response data via reverse dosimetry.

In the reverse dosimetry approach, the PBTK model was used in reverse order to identify the retrorsine dose required to obtain a specific retrorsine concentration in the liver. This was achieved by setting in vitro concentrations $C_\textrm{in vitro}$ equal to maximum concentrations in the lumped vascular/interstitial (superscript vi) liver space $C^{\textrm{vi}}_{\textrm{max},\textrm{liv}}$ and correcting for protein binding (Eq. 3; fraction unbound in experimental assay $\textrm{fu}_\textrm{in vitro}$; fraction unbound in blood plasma $\textrm{fuP}$).

3 $$\begin{aligned} \textrm{fu}_\textrm{in vitro}\cdot C_\textrm{in vitro} =&\, \textrm{fuP}\cdot C^{\textrm{vi}}_{\textrm{max},\textrm{liv}} \end{aligned}$$

Since the incubation medium used in the present study for the in vitro cytotoxicity assay was serum free, absence of protein binding in vitro was assumed ($\textrm{fu}_\textrm{in vitro}$=1). This assumption was supported by the prediction of non-specific binding to hepatocytes yielding $\textrm{fu}_\textrm{in vitro}$ of 0.994 (Austin et al. 2005). The in vitro response ’cell viability’ was set equal to the in vivo response ’liver integrity’. A decrease of 5% in ’liver integrity’ means that liver toxicity is increased by 5%.

Predicted in vivo dose-response data were then used to identify a benchmark dose (BMD) confidence interval for mouse and rat. BMD analysis was performed for a 5% benchmark response (change in mean response compared to controls) using the web application https://r4eu.efsa.europa.eu/app/bmd (Accessed 20 March 2022) (Varewyck et al. 2017). The application uses a set of given parametric dose-response models to describe the data. Model averaging was performed to derive a 90% confidence interval around the BMD according to the EFSA guidance document (EFSA 2017b). The confidence interval was reported as lower bound (BMDL$_{5}$) and upper bound (BMDU$_{5}$).

## Results

### Kinetic data collected from animal studies

We identified six animal studies performed with mice and/or rats that reported in vivo measurements of retrorsine and/or its hepatic metabolites DHR:DNA, DHR:PROT and DHR:GSH [Table S2, Chu and Segall (1991), Li et al. (2022), White (1977), Yang et al. (2017, 2018), Zhu et al. (2017)]. With respect to the routes of administration, intraperitoneal (i.p.) administration was most abundant followed by per oral (p.o.) and intravenous (i.v.) administration of retrorsine solutions. Retrorsine was administered at acutely toxic doses ranging from 5 to 70 mg/kg bodyweight. Three of the most recent studies were (partly) considered as independent evaluation data (see Table S2 column ’Function’). Retrorsine levels at 12 h and 24 h as reported in Li et al. (2022) were excluded from the compiled dataset, as graphical extraction of retrorsine levels near 0 $\upmu$g/mL from a linear scale was not possible with sufficient accuracy.

### Characterization of retrorsine toxicokinetics

#### Oral absorption

Retrorsine is moderately fast absorbed from the gut tissue as indicated by the intestinal absorption rate constant $k_{\textrm{a}}$ (1/h) of 0.910 [0.581, 3.51] estimated from mouse data. The fraction absorbed from the gut $F_{\textrm{a}}$ predicted as 78.3% shows that a high percentage of retrorsine is passing the intestinal barrier. The prediction of $F_{\textrm{a}}$ was based on the reported correlation between $F_{\textrm{a}}$ and in vitro Caco-2 permeability $P_{\textrm{app}, \textrm{A}\rightarrow \textrm{BL}}$ (Skolnik et al. 2010). Caco-2 permeabilities $P_{\textrm{app}, \textrm{A}\rightarrow \textrm{BL}}$ and $P_{\textrm{app}, \textrm{BL}\rightarrow \textrm{A}}$ are moderate as determined in the bidirectional Caco-2 assay (Fig. S2). The low efflux ratio (ER = $P_{\textrm{app}, \textrm{A}\rightarrow \textrm{BL}}$/$P_{\textrm{app}, \textrm{BL}\rightarrow \textrm{A}}$ ) of $1.75 \pm 0.952$ indicates that retrorsine has high intestinal permeability and is not a substrate to intestinal efflux transporters.

#### Protein binding and tissue distribution

We previously measured protein binding of retrorsine in human blood plasma using rapid equilibrium dialysis (Haas et al. 2019). From this, we determined a fraction unbound in blood plasma (fuP) of 60.0%. The in silico prediction of 63.8% using the ADMET Predictor (GastroPlus$^\text {TM}$, SimulationsPlus (2019)) is in line with the experimentally determined fuP.

In terms of tissue distribution, retrorsine is most weakly distributed into adipose ($K_{\textrm{adi}}$ = 0.184) and bone ($K_{\textrm{bon}}$ = 0.602) and most strongly distributed into muscle, brain and gut tissue ($K_{\textrm{mus}}\approx K_{\textrm{bra}}\approx K_{\textrm{gut}}\approx$ 1) according to the Rodgers and Rowland model. All predicted tissue-to-plasma partition coefficients $K_{\textrm{tis}}$ are listed in Table S1.

#### Hepatic elimination based on the extended clearance model

**Fig. 2:**
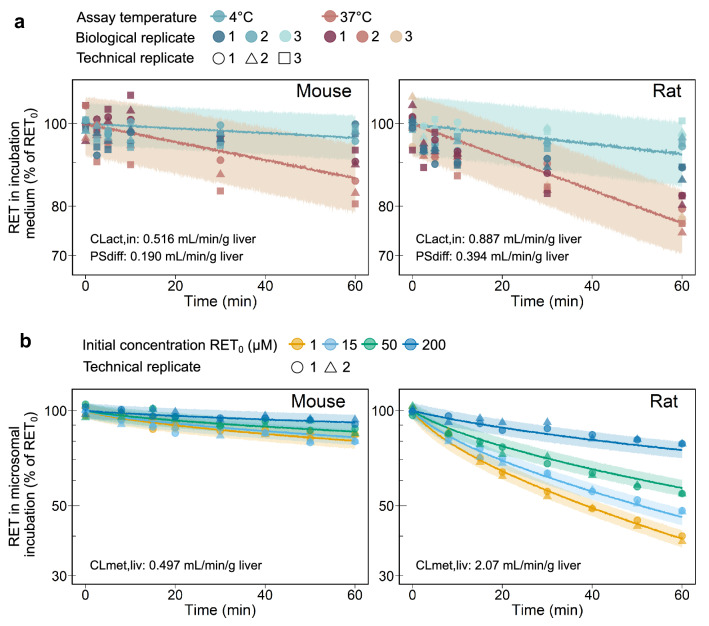
Temperature-dependent retrorsine depletion-time profiles in incubations of primary mouse and rat hepatocytes (**a**). Medium loss assays were performed with 0.7 $\upmu$M of retrorsine (RET$_0$) either at 4 °C or at 37 °C. Observed data (*n*=2 (mouse) or n=3 (rat) biological replicates, each assessed with *n*=3 technical replicates) were described by a monoexponential decay model (Eq. S23). Median (solid lines) and 5–95% interpercentile range (shaded areas) are based on 1000 Monte Carlo simulations. In vivo liver active uptake clearance $\textrm{CL}_{\textrm{act},\textrm{in}}$ and passive influx diffusion flow rate $\textrm{PS}_{\textrm{diff}}$ were predicted for non-saturating conditions (Eqs. S24–S27). Concentration-dependent retrorsine depletion-time profiles in liver microsomal incubations of mouse and rat (**b**). Microsomal assays were performed with 1, 15, 50 or 200 $\upmu$M of retrorsine (RET$_0$). Observed data (*n*=2 technical replicates using microsomes of pooled livers) were described by an end-product inhibition model (Eqs. S28–S31). Median (solid lines) and 5–95% interpercentile range (shaded areas) are based on 1000 Monte Carlo simulations. In vivo liver metabolic clearance $\textrm{CL}_{\textrm{met}, \textrm{liv}} = V_{\textrm{max},\textrm{liv}}/K_{\textrm{M},\textrm{liv}}$ was predicted for the case of linear kinetics ($\textrm{RET}<< K_{\textrm{M},\textrm{liv}}$)

In the extended clearance model an interplay of the four processes hepatic uptake, efflux, metabolism and biliary excretion is considered to impact hepatic elimination. Regarding hepatic uptake, retrorsine permeates the sinusoidal membrane via passive diffusion and via carrier-mediated active uptake. The predicted active uptake clearance $\textrm{CL}_{\textrm{act},\textrm{in}}$ (mL/min/g liver) of 0.516 in mouse and 0.887 in rat reveals that active uptake is the primary route of hepatic membrane permeation. Passive diffusion contributes to a lower extent to hepatic membrane permeation as shown by the predicted passive influx diffusion flow rate $\textrm{PS}_{\textrm{diff}}$ (mL/min/g liver) of 0.190 in mouse and 0.394 in rat. $\textrm{CL}_{\textrm{act},\textrm{in}}$ and $\textrm{PS}_{\textrm{diff}}$ were derived from in vitro medium loss assays, in which primary hepatocytes were incubated under physiological (37 °C) and non-physiological (4 °C), non-saturating conditions. Time-dependent retrorsine depletion in the incubation medium was best described by the monoexponential decay model (Eq. S23, Fig. 2a, Table 1).

Retrorsine taken up into the liver cellular space is metabolized and excreted via the bile. Hepatic metabolism of retrorsine is highly species-specific as shown by the 4-fold higher liver metabolic clearance $\textrm{CL}_{\textrm{met}, \textrm{liv}}$ (mL/min/g liver, ratio of $V_{\textrm{max},\textrm{liv}}$ and $K_{\textrm{M},\textrm{liv}}$) in the rat (2.07) compared to the mouse (0.497). Michaelis-Menten constant $K_{\textrm{M},\textrm{liv}}$ and maximum reaction velocity $V_{\textrm{max},\textrm{liv}}$ of liver metabolism were predicted from in vitro liver microsomal assays. Concentration- and time-dependent retrorsine depletion in microsomal incubations was best described by the end-product inhibition model (Eqs. S28–S31, Fig. 2b, Table 1). In the end-product inhibition model we assumed irreversible inhibition of biotranformation enzymes by reactive retrorsine metabolites as demonstrated in vitro with CYP3A4 (Dai et al. 2010). The product concentration IC$_{50}$, at which retrorsine biotransformation was inhibited half-maximal, was estimated 5-fold smaller in mouse compared to rat microsomes.

Excretion of retrorsine into the bile plays a minor role as indicated by the low rat biliary clearance CL$_{\textrm{bile}}$ (mL/min/g liver) of 0.101 [0.0878, 0.118] estimated from rat in vivo bile levels. For the mouse, the absence of biliary excretion was assumed (no bile data available). Active sinusoidal efflux of retrorsine was assumed negligible in both species.

According to the extended clearance model (Eq. 2), overall liver clearance $\textrm{CL}_{\textrm{liv}}$ (mL/min/kg bodyweight) was similar in mouse and rat with 14.8 and 14.6, respectively. $\textrm{CL}_{\textrm{liv}}$ makes up about 70% (mouse) and 62% (rat) of the total clearance of retrorsine.

**Table 1:** Parameter estimates of the monoexponential decay model (medium loss assay, Eq. S23) and the end-product inhibition model (microsomal assay, Eqs. S28–S31)

Parameter	Unit	Posterior mode [95% credible interval]			
		Mouse		Rat	
Monoexponential decay model					
$\lambda _{4^\circ \text {C}}$	1/h	0.0401	[0.0140, 0.0617]	0.0832	[0.0518, 0.114]
$\lambda _{37^\circ \text {C}}$	1/h	0.149	[0.111, 0.185]	0.270	[0.240, 0.301]
End-product inhibition model					
$\textrm{IC}_{50}$	$\upmu$M	0.0400	[0.0111, 0.152]	0.197	[0.110, 0.273]
$V_{\textrm{max},\textrm{liv}, \textrm{in vitro}}$	$\upmu$M/min	0.586	[0.349, 1.18]	1.95	[1.64, 2.41]
$V_{\textrm{max},\textrm{liv}}$ $^\textrm{a}$	nmol/min/g liver	27.6	[16.4, 55.5]	91.7	[77.1, 113]
$K_{\textrm{M},\textrm{liv}}$	$\upmu$M	55.4	[35.4, 106]	44.3	[37.2, 51.3]

$^\textrm{a}$Predicted by in vitro-to-in vivo extrapolation (Eq. S32)

#### Extrahepatic elimination

Extrahepatic elimination pathways considered in the PBTK model were renal excretion and intestinal metabolism. Retrorsine accumulation in urine was best described by the PBTK model when renal clearance CL$_{\textrm{uri}}$ was expressed as a product of fuP and glomerular filtration rate. This yielded CL$_{\textrm{uri}}$ (mL/min/kg bodyweight) of 4.80 for mouse and rat. Thereby, glomerular filtration was considered as the predominant mechanism of retrorsine renal excretion, while active secretion and tubular reabsorption were assumed to play a minor role. Predicted CL$_{\textrm{uri}}$ makes up about 23% (mouse) or 20% (rat) of total retrorsine clearance.

Intestinal metabolic clearance $\textrm{CL}_{\textrm{met},\textrm{gut}}$ (mL/min/kg bodyweight, ratio of $V_{\textrm{max},\textrm{gut}}$ and $K_{\textrm{M},\textrm{gut}}$) was determined as 2.73 (mouse) and 7.58 (rat). The Michaelis-Menten constant $K_{\textrm{M},\textrm{gut}}$ was assumed to be equal to that of liver metabolism and the maximum reaction velocity $V_{\textrm{max},\textrm{gut}}$ was assumed to be reduced by factor 10 compared to liver metabolism (Galetin and Houston 2006). Intestinal metabolism contributes with 8% (mouse) or 18% (rat) to total clearance of retrorsine in the PBTK model.

### Training and evaluation of the PBTK model

**Fig. 3:**
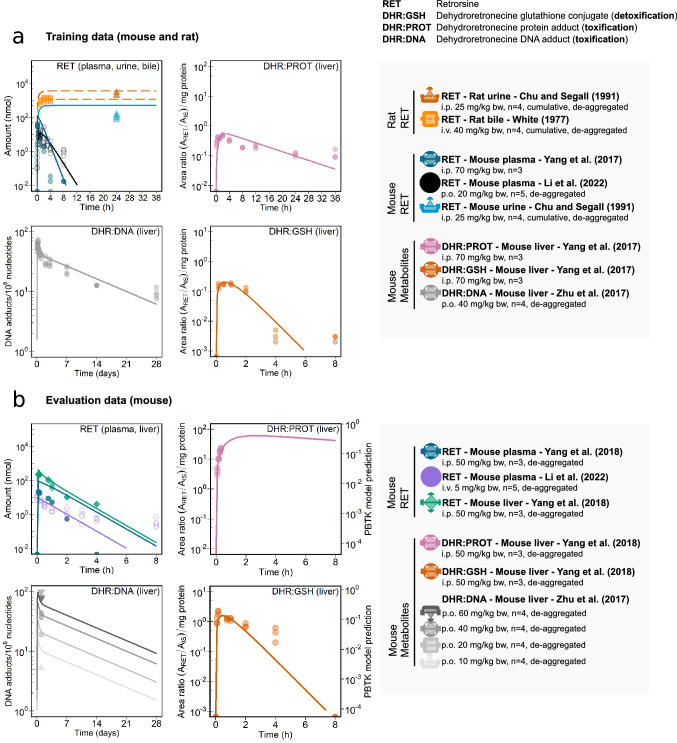
Kinetic data and PBTK model predictions of tissue amount-time profiles of retrorsine and its hepatic metabolites DHR:GSH, DHR:PROT, DHR:DNA in mouse and/or rat. Training data were used for PBTK model development (**a**). Evaluation of PBTK model predictivity was performed with independent mouse data (**b**). Kinetic in vivo studies with retrorsine are summarized in Table S2. Retrorsine was administered intravenously (i.v.), intraperitoneally (i.p.) or orally (p.o.) with doses ranging from 5 to 70 mg/kg bodyweight. Symbols and colours link to the legend, where an overview of species, tissue, study author (year), administration route, dose and number *n* of animals is provided. When indicated, summary data were ’de-aggregated’ as described in Hethey et al. (2021). Most kinetic data were reported with different units in original studies. For the modeling procedure kinetic data were standardized into the common base unit ’amount of substance’ (nmol) (Supplementary Information ’Pre-processing of in vivo kinetic data’). Note: Hepatic metabolites DHR:GSH and DHR:PROT were reported as mass spectrometric peak area ratio of analyte to internal standard in original studies. PBTK model predictions of those relative quantities are marked by a 2$\text {nd}$ y-axis in the evaluation dataset

All of the ten unknown PBTK model parameters were identifiable in the MCMC analysis and were estimated with low uncertainty (Table S1, Fig. S3). Overall, levels of retrorsine and hepatic retrosine metabolites were well described by PBTK model predictions. A graphical representation of the model fits is shown in Fig. 3a (colors relate to figure legend). Retrorsine amount in mouse plasma (i.p. administration, dark blue) and urine (light blue) was slightly overpredicted, while retrorsine amount in mouse plasma (p.o.administration, black), as well as in rat bile and urine (orange tones) was well described.

Predictions of DHR:DNA (grey) successfully described their biexponential depletion pattern. The biphasic kinetics of DHR:DNA degradation were explained by compartmentalization of DNA damage and repair (Talaska et al. 1996; Zhu et al. 2017). DHR:DNA that are removed much slower as indicated by the second depletion phase were assumed to reside in repair resistant compartments as was observed with DNA adducts from other compounds.

DHR:PROT (pink) and DHR:GSH (red) depletion kinetics were best described by a monoexponential decay model. For the latter two metabolites, the quality of PBTK model predictions was adequate for early time points but declined at later time points. Residual plots are provided in Fig. S4.

Results of the PBTK model evaluation are depicted in Fig. 3b. Retrosine kinetics in mouse liver (green) agreed to the model predictions, while the model was not trained with any liver data. Again, the amount of retrosine in plasma after i.p. administration (dark blue) was moderately overpredicted, while the retrorsine amount in plasma after i.v. administration (purple) was slightly underestimated for late time points. The predicted dose-dependent formation of DHR:DNA (grey tones) matched the observed data. Of note, DHR:PROT (pink) and DHR:GSH (red) were reported as relative quantities in original studies (mass spectrometric peak area ratio of analyte to internal standard) (Yang et al. 2017, 2018). The PBTK model successfully predicted the kinetic course of both metabolites, while the prediction of relative quantities was shifted upwards by factor 12 (DHR:GSH) or factor 150 (DHR:PROT) compared to observed data as marked by the second y-axis.

### PBTK model prediction after oral dose

**Fig. 4:**
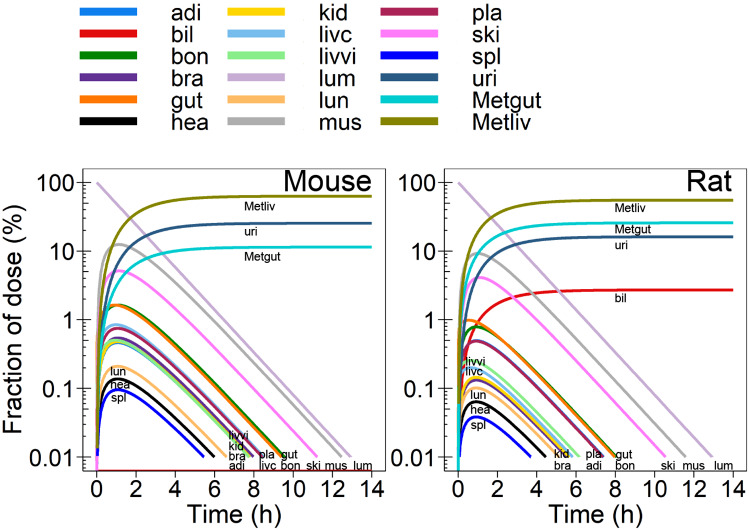
Predicted disposition pattern [fraction of administered dose (%)] in mouse and rat after single oral dose of 1 mg/kg bodyweight retrorsine. Note: bil, uri, Metgut and Metliv are given as a cumulative fraction of dose (%). Abbreviations: adi adipose; bil bile; bon bone; bra brain; gut gut tissue; hea heart; kid kidneys; livc liver cellular space; livvi liver vascular/interstitital space; lum gut lumen; lun lungs; mus muscle; pla blood plasma; ski skin; spl spleen; uri urine; Metgut total gut metabolites; Metliv total liver metabolites

The time course of the fraction of administered dose predicted in mouse and rat after single oral administration of retrorsine is shown in Fig. 4. Following rapid absorption from the gut lumen, the highest retrorsine fractions were predicted in muscle (12% mouse; 9% rat) and skin (5% mouse; 4% rat), while the lowest retrorsine fractions were predicted in spleen, heart and lungs ($\le$ 0.2% mouse; $\le$ 0.1% rat). In blood plasma, retrorsine peaked to 0.7% (mouse) or 0.5% (rat) of administered dose.

Elimination of retrorsine from tissues was slightly slower in mouse compared to rat. After 12.5 h in mouse and 12 h in rat, 99.99% of retrorsine were eliminated from the body. Liver metabolism accounted for 63% (mouse) or 55% (rat) of retrorsine elimination, while 11% (mouse) or 26% (rat) of retrorsine were metabolized by the gut tissue. 25% (mouse) or 16% (rat) of the dose were excreted renally. Biliary excretion contributed to approximately 3% of retrorsine elimination in rat (assumed absent in mouse).

### Prediction of acute liver toxicity after oral dose

**Fig. 5:**
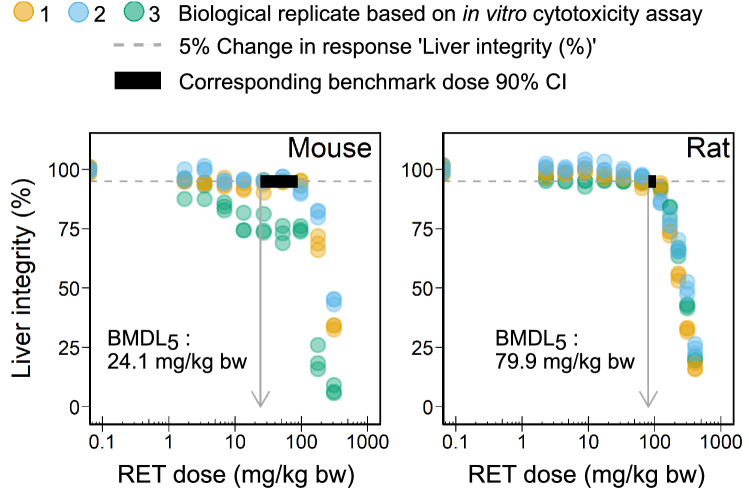
In vivo dose-response curve for acute liver toxicity of retrorsine in mouse and rat predicted from in vitro data by reverse dosimetry using the evaluated PBTK model. Retrorsine (RET) dose is given in mg/kg bodyweight and response is expressed as liver integrity (%). A 5% reduction in liver integrity equates to a 5% increase of liver toxicity. Colors relate to biological replicates originating from in vitro cytotoxicity assays with primary mouse and rat hepatocytes. Benchmark dose (BMD) 90% confidence intervals (black bar) for a 5% change of response (dashed line) were derived for mouse and rat by BMD analysis using the web application https://r4eu.efsa.europa.eu/app/bmd (Accessed 20 March 2022, Varewyck et al. (2017)). The grey arrow represents the lower bound of the confidence interval (BMDL$_{5}$)

In vivo dose-response data were predicted from in vitro cytotoxicity data via PBTK model-based reverse dosimetry. In vitro determined IC$_{50}$ values for retrorsine cytotoxicity in primary hepatocytes were 148 $\upmu$M (mouse) and 153 $\upmu$M (rat) (Eq. S36, Fig. S5). The observed high inter-individual variability for the mouse originates from the varying sensitivity of primary mouse hepatocytes towards retrorsine. The predicted BMD confidence interval for acute liver toxicity of retrorsine was broader in mice with 24.1–88.5 mg/kg bodyweight compared to rats with 79.9–104 mg/kg bodyweight (Fig. 5). An orally administered retrorsine dose within this interval provokes a 5% decrease in the response ’liver integrity’. Detailed reports of the BMD analysis are provided in Fig. S6–S8.

## Discussion

Current risk assessment of hepatotoxic PA is facing data gaps for many individual PA regarding their toxicokinetics, metabolic activation and carcinogenic potency (EFSA 2017a). In this study, we presented a novel PBTK model predicting the species-specific toxicokinetics of the PA retrorsine in mouse and rat. Therefore, we comprehensivley characterized the toxicokinetics of retrorsine by means of in vitro assays and in vitro-to-in vivo extrapolation. Dose-response studies enable the derivation of a benchmark dose for risk assessment of retrorsine’s liver toxicity are not available in animals and humans. Here, we demonstrated a PBTK model-based approach to predict in vivo acute liver toxiciy of retrorsine based on in vitro toxicity studies with primary hepatocytes.

Retrorsine was selected as model PA because time-dependent in vivo data in plasma, liver, urine or bile of mouse and/or rat were available. As retrorsine is a known substrate of the sinusoidal influx transporters OCT1 and NTCP (Enge et al. 2021; Tu et al. 2014), we included a mechanistic liver model, which takes into account the role of hepatic transport additionally to metabolism and biliary excretion (Patilea-Vrana and Unadkat 2016; Sirianni and Pang 1997). This constitutes a major step forward in the prediction of toxicokinetics compared to previously reported PBTK models of PA. In addition, the presented PBTK model for the first time includes a simplified model of retrorsine liver metabolism, as in vivo measurements of DNA adducts, protein adducts and GSH conjugates were available from animal studies. Intestinal metabolism and renal excretion were included as elimination routes additionally to hepatic elimination.

Liver microsomal assays showed notable species differences in metabolic clearance of retrorsine with a 4-fold higher clearance in the rat compared to the mouse. Medium loss assays with primary hepatocytes revealed a 1.7-fold higher hepatic active uptake clearance of retrorsine in rat compared to mouse. This observed species-specific difference correlates with measured OCT1 levels in hepatocytes, which displayed a similar fold change in rat compared to mouse (Morse et al. 2021). Of note, the active uptake clearance describes the phenomenological uptake by all transporters that retrorsine is a substrate including OCT1 and NTCP. Individual kinetics of distinct uptake transporters as well as saturation of transport were not included in the PBTK model. In mouse and rat, the liver was predicted to act as a sink, since the sum of retrorsine elimination via metabolism and biliary excretion was notably higher compared to sinusoidal efflux. In the rat, transporter-mediated hepatic uptake was identified as the rate-limiting step in hepatic clearance, as it was twofold smaller than metabolic elimination. In the mouse, both active uptake and metabolism were determined to be rate-limiting. Estimated rat biliary clearance was about one-tenth of rat hepatic active uptake clearance. This points towards a minor role of retrorsine biliary excretion and is in line with reported in vitro substrate activity towards biliary efflux transporters. Retrorsine showed only weak or no interaction with P-glycoprotein (MDR1), breast cancer resistance protein (BCRP) and multidrug and toxin extrusion protein 1 (MATE1) (Tu et al. 2014).

Of note, evaluation of the presented PBTK model was not only performed with in vivo blood plasma data as reported before for other PA but also with in vivo liver data. The good predictivity of retrorsine kinetics in the liver qualified the PBTK model for its application in the reverse dosimetry approach. We exemplarily performed reverse dosimetry for the toxicological endpoint acute liver toxicity. Therefore, we translated liver toxicity data (toxicodynamics) obtained from primary hepatocytes as in vitro model into the in vivo situation by taking into account the species-specific toxicokinetics. Based on the in vivo dose-response data predicted for oral dosing of retrorsine, we derived a benchmark dose (BMD) confidence interval for acute liver toxicity in mouse and rat. The predicted lower bound BMDL$_5$ was 3-fold higher for rats compared to mice. It remains questionable, if this reflects a true species difference in retrorsine toxicity or if the difference originates from the high inter-individual variability observed in the cytotoxicity assay with mouse primary hepatocytes. BMD analysis omitting the third mouse biological replicate would yield a BMDL$_5$ of 83.3 mg/kg bodyweight, which is similar to that predicted for rats.

A proof-of-principle that a BMD derived from in vitro cytotoxicity data by PBTK model-based reverse dosimetry is a good approximation of in vivo acute liver toxicity data has been provided for the PA lasiocarpine, riddelliine and monocrotaline (Chen et al. 2018; Suparmi et al. 2020). A comparison of retrorsine BMD predictions to established points of departure for risk assessment was not possible due to non-availability of dose-response studies. However, LD$_{50}$ values after single intravenous (i.v.) injection of retrorsine were reported as 59 mg/kg bodyweight in mouse and 38 mg/kg bodyweight in rat (Mattocks 1986; Merz and Schrenk 2016). In general, we would expect a dose provoking a 5% increase in liver toxicity to be smaller than a dose inducing the death of 50% of animals. To test this hypothesis, we additionally derived BMDL$_5$ after i.v. administration of retrorsine: they are 25-fold (mouse) or 8-fold (rat) smaller than reported LD$_{50}$ values.

In comparison to other PA, our predicted rat BMDL$_{5}$ of retrorsine of 79.9 mg/kg bodyweight is 3.5-fold and 16-fold higher than predicted BMDL$_5$ of lasiocarpine and riddelliine (23.0 and 4.9 mg/kg bodyweight) (Chen et al. 2018). Of note, the IC$_{50}$ value of retrorsine of 153 $\upmu$M determined from in vitro cytotoxicity in rat hepatocytes was 14-fold and 24-fold higher compared to the IC$_{50}$ of lasiocarpine and riddelliine (10.9 and 6.3 $\upmu$M). Translation of toxicodynamics from in vitro cytotoxicity assays to the in vivo situation by integration of toxicokinetics hence alters the distance in toxicodynamics between PA.

In our approach, data obtained from primary cell culture were extrapolated to the in vivo situation under the assumption that the susceptibility of primary cultured hepatocytes towards retrorsine is similar to that of hepatocytes in vivo. However, previous studies showed that the susceptibility towards toxins as well as the expression of metabolizing enzymes and hepatic transporters of cultured hepatocytes can be affected upon isolation (Godoy et al. 2013). With regard to culture conditions, Gao et al. (2020) found that in cytotoxicity testing short pre-incubation times of hepatocytes (3 h vs. 24 h) increased the sensitivity of rat hepatocytes towards retrorsine. An increased sensitivity was also reported for long incubation times with the test compound (48 h vs. 24 h).

Here, we derived an IC$_{50}$ value of 153 $\upmu$M retrorsine in rat hepatocytes with 3 h pre-incubation and 48 h incubation. Our findings were comparable to the IC$_{50}$ value of 163 $\upmu$M retrorsine with the same pre-incubation time, but a shorter incubation time of 24 h (Gao et al. 2020). However, the strong increase in cytotoxicity (IC$_{50}$ of 19 $\upmu$M) after 48 h of incubation as in Gao et al. (2020) was not observed in this study. An increase in cytotoxicity would mean a strong reduction of predicted BMDL$_5$ values for acute liver toxicity of retrorsine. To a certain extent, differences in IC$_{50}$ can be reasoned in the choice of the mathematical model to describe the experimental data. In this study, a sigmoidal inhibition model (Eq. S36) was used for the derivation of IC$_{50}$.

Next to the culture conditions, the selection of the in vitro liver cell model is affecting the outcome of the BMD prediction. Enge et al. (2021) reported that HepaRG cells incubated with 250 $\upmu$M retrorsine had a viability of 55% compared to the solvent control (24 h incubation, IC$_{50}$ not provided). Viability of CYP3A4-expressing sinusoidal endothelial cells was reduced to 61% with 600 $\upmu$M retrorsine (48 h incubation, IC$_{50}$ not provided, Lu et al. (2020)). Compared to HepaRG and sinusoidal endothelial cells, rat and mouse hepatocytes were more sensitive towards retrorsine treatment. In comparison to HepG2 cells (IC$_{50}$ = 73 $\upmu$M, 48 h incubation, Rutz et al. (2020)), rat and mouse hepatocytes appear less sensitive with regard to the results of this study, while more sensitive relating to the findings of Gao et al. (2020). Based on this outline of available literature, we conclude that primary hepatocytes are a sensitive cell model appropriate for hepato-cytotoxicity testing and BMD derivation.

A limitation of this study was the prediction of protein adducts (DHR:PROT), biomarkers of acute hepatotoxicity, and GSH conjugates (DHR:GSH), which are generally regarded as detoxification metabolites. Model evaluation showed that the PBTK model qualitatively, but not quantitatively predicted the toxicokinetics of DHR:PROT and DHR:GSH. In vivo kinetic measurements of both metabolites were reported in original studies with relative units (Yang et al. 2017, 2018). If absolute quantities of both metabolites were available, the ratio of DHR:PROT to DHR:GSH could serve as a measure to quantify retrorsine toxification versus detoxification.

With respect to DNA adducts, a biomarker for PA expoure, the prediction of toxicokinetics needs to be clearly separated from the prediction of a genotoxic response. The presented PBTK model describes and predicts the toxicokinetics of retrorsine-derived DNA adducts including the observable biexponential depletion kinetics. The PBTK model has no toxicodynamic part incorporated and does not hold implications on chronic endpoint predictions. As such, the link between DNA adduct concentration and genotoxic response was not informed in this study. Perspectively, the PBTK model can aid in the prediction of chronic effects by translating concentration-response data from genotoxicity read-outs into in vivo dose-response data via reverse dosimetry (Chen et al. 2019).

Due to the generic nature of the PBTK approach, the presented PBTK model can also be applied to other hepatotoxic PA by replacing physico-chemical/biochemical model parameters, or can be used for extrapolation to other relevant species (e.g. humans or farm animals) by replacing physiology-specific model parameters.

In conclusion, we demonstrated a combined in vitro and in silico approach for the development of a PBTK model of retrorsine, which takes into account the role of hepatic transport additionally to liver metabolism. Using the example of the toxicological endpoint acute liver toxicity, we used the evaluated PBTK model to predict in vivo hepatotoxic dose levels and derived BMD values in mouse and rat. Our results underline the importance of toxicokinetic differences between PA and between species that need to be taken into account when extrapolating toxicodynamics to the in vivo situation in animals and also humans.

## Supplementary Information

Below is the link to the electronic supplementary material.

Supplementary file1 (PDF 5247 KB)

## Data Availability

All data, material and code are available in the GitHub repository https://github.com/al901010/Supplement_PBTK_Retrorsine_Mouse_Rat.
