# Supplementary material for: PBTK modeling of the pyrrolizidine alkaloid retrorsine to predict liver toxicity in mouse and rat
Source: Arch Toxicol. 2023 Mar 11;97(5):1319–33. doi: 10.1007/s00204-023-03453-z (PMC10110657; doi:10.1007/s00204-023-03453-z)
Supplement: Supplementary file 1 — Supplementary file1 (PDF 5247 KB) [file 204_2023_3453_MOESM1_ESM.pdf]

Archives of Toxicology

PBTK modeling of the pyrrolizidine alkaloid retrorsine to predict liver toxicity in mouse and rat

Supplementary document

Anja Lehmann[1,2] · Ina Geburek[1] · Anja These[1] · Stefanie Hessel-Pras[1] · Jan G. Hengstler[3] · Wiebke Albrecht[3] · Hans Mielke[1] · Christine Müller-Graf[1] · Xiaojing Yang[4] · Charlotte Kloft[2] · Christoph Hethey[1]

Corresponding author: Hans Mielke
Tel.: +49-30-18412-23303
E-mail: hans.mielke@bfr.bund.de

[1] German Federal Institute for Risk Assessment (BfR), Max-Dohrn-Str. 8-10, 10589 Berlin, Germany

[2] Department of Clinical Pharmacy and Biochemistry, Institute of Pharmacy, Freie Universitaet Berlin, 12169 Berlin, Germany

[3] Leibniz Research Centre for Working Environment and Human Factors (IfADo), Technical University of Dortmund, 44139 Dortmund, Germany

[4] Wuya College of Innovation, Shenyang Pharmaceutical University, Shenyang, Liaoning 110016, P. R. China

PBTK Model Ordinary Differential Equations

The following ordinary differential equations describe the change of amount of substance (mol) of retrorsine or its metabolites over time t (h) in the tissues (tis) of the PBTK model indicated below. All model parameters are listed in Table S1.

Retrorsine (RET) in peritoneum (for intraperitoneal administration):

$$\frac{d\mathrm{RET}_{\mathrm{per}}(t)}{dt} = -k_{\mathrm{per}} \cdot \mathrm{RET}_{\mathrm{per}}(t) \tag{S1}$$

Retrorsine in gut lumen (for oral administration):

$$\frac{d\mathrm{RET}_{\mathrm{lum}}(t)}{dt} = -k_{\mathrm{a}} \cdot F_{\mathrm{a}} \cdot \mathrm{RET}_{\mathrm{lum}}(t) \tag{S2}$$

Retrorsine in venous blood (for intravenous administration):

$$\frac{d\mathrm{RET}_{\mathrm{ven}}(t)}{dt} = \sum_{\mathrm{tis}} Q_{\mathrm{tis}} \cdot \frac{\mathrm{RET}_{\mathrm{tis}}(t)}{V_{\mathrm{tis}} \cdot K_{\mathrm{tis}}} + Q_{\mathrm{liv}} \cdot K_{\mathrm{liv}}^{\mathrm{vas:vi}} \cdot \frac{\mathrm{RET}_{\mathrm{liv}}^{\mathrm{vi}}(t)}{V_{\mathrm{liv}}^{\mathrm{vi}}} - Q_c \cdot \frac{\mathrm{RET}_{\mathrm{ven}}(t)}{V_{\mathrm{ven}}} \quad \mathrm{tis} = \mathrm{adi}, \mathrm{bon}, \mathrm{bra}, \mathrm{hea}, \mathrm{kid}, \mathrm{mus}, \mathrm{ski} \tag{S3}$$

Retrorsine in arterial blood:

$$\frac{d\mathrm{RET}_{\mathrm{art}}(t)}{dt} = Q_c \cdot \left(\frac{\mathrm{RET}_{\mathrm{lun}}(t)}{V_{\mathrm{lun}} \cdot K_{\mathrm{lun}}} - \frac{\mathrm{RET}_{\mathrm{art}}(t)}{V_{\mathrm{art}}} \right) \tag{S4}$$

Retrorsine in lungs:

$$\frac{d\mathrm{RET}_{\mathrm{lun}}(t)}{dt} = Q_c \cdot \left(\frac{\mathrm{RET}_{\mathrm{ven}}(t)}{V_{\mathrm{ven}}} - \frac{\mathrm{RET}_{\mathrm{lun}}(t)}{V_{\mathrm{lun}} \cdot K_{\mathrm{lun}}} \right) \tag{S5}$$

Retrorsine in adipose, bone, brain, heart, muscle, skin or spleen:

$$\frac{d\mathrm{RET}_{\mathrm{tis}}(t)}{dt} = Q_{\mathrm{tis}} \cdot \left(\frac{\mathrm{RET}_{\mathrm{art}}(t)}{V_{\mathrm{art}}} - \frac{\mathrm{RET}_{\mathrm{tis}}(t)}{V_{\mathrm{tis}} \cdot K_{\mathrm{tis}}} \right) \quad \mathrm{tis} = \mathrm{adi}, \mathrm{bon}, \mathrm{bra}, \mathrm{hea}, \mathrm{mus}, \mathrm{ski}, \mathrm{spl} \tag{S6}$$

Retrorsine in kidneys:

$$\frac{d\mathrm{RET}_{\mathrm{kid}}(t)}{dt} = Q_{\mathrm{kid}} \cdot \left(\frac{\mathrm{RET}_{\mathrm{art}}(t)}{V_{\mathrm{art}}} - \frac{\mathrm{RET}_{\mathrm{kid}}(t)}{V_{\mathrm{kid}} \cdot K_{\mathrm{kid}}} \right) - \mathrm{fuP} \cdot \mathrm{GFR} \cdot \frac{\mathrm{RET}_{\mathrm{kid}}(t)}{V_{\mathrm{kid}}} \tag{S7}$$

Retrorsine in urine:

$$\frac{d\mathrm{RET}_{\mathrm{uri}}(t)}{dt} = \mathrm{fuP} \cdot \mathrm{GFR} \cdot \frac{\mathrm{RET}_{\mathrm{kid}}(t)}{V_{\mathrm{kid}}} \tag{S8}$$

Retrorsine in in gut tissue:

$$\begin{aligned}\frac{d\mathrm{RET}_{\mathrm{gut}}(t)}{dt} &= k_{\mathrm{a}} \cdot F_{\mathrm{a}} \cdot \mathrm{RET}_{\mathrm{lum}}(t) + Q_{\mathrm{gut}} \cdot \left(\frac{\mathrm{RET}_{\mathrm{art}}(t)}{V_{\mathrm{art}}} - \frac{\mathrm{RET}_{\mathrm{gut}}(t)}{V_{\mathrm{gut}} \cdot K_{\mathrm{gut}}}\right) \\ &\quad - \frac{V_{\mathrm{max,gut}}}{K_{\mathrm{M,gut}} + \mathrm{fu}_{\mathrm{gut}} \cdot \frac{\mathrm{RET}_{\mathrm{gut}}(t)}{V_{\mathrm{gut}}}} \cdot \mathrm{fu}_{\mathrm{gut}} \cdot \frac{\mathrm{RET}_{\mathrm{gut}}(t)}{V_{\mathrm{gut}}}\end{aligned} \tag{S9}$$

Retrorsine in liver vascular/interstitial (superscript vi; also termed extracellular) space:

$$\begin{aligned}\frac{d\mathrm{RET}^{\mathrm{vi}}_{\mathrm{liv}}(t)}{dt} &= k_{\mathrm{per}} \cdot V_{\mathrm{per}} \cdot \mathrm{RET}_{\mathrm{per}}(t) + (Q_{\mathrm{liv}} - Q_{\mathrm{spl}} - Q_{\mathrm{gut}}) \cdot \frac{\mathrm{RET}_{\mathrm{art}}(t)}{V_{\mathrm{art}}} + Q_{\mathrm{spl}} \cdot \frac{\mathrm{RET}_{\mathrm{spl}}(t)}{V_{\mathrm{spl}} \cdot K_{\mathrm{spl}}} \\ &\quad + Q_{\mathrm{gut}} \cdot \frac{\mathrm{RET}_{\mathrm{gut}}(t)}{V_{\mathrm{gut}} \cdot K_{\mathrm{gut}}} - Q_{\mathrm{liv}} \cdot K^{\mathrm{vas:vi}}_{\mathrm{liv}} \cdot \frac{\mathrm{RET}^{\mathrm{vi}}_{\mathrm{liv}}}{V^{\mathrm{vi}}_{\mathrm{liv}}} - (\mathrm{CL}_{\mathrm{act,in}} + \mathrm{PS}_{\mathrm{diff}}) \cdot K^{\mathrm{int,u:vi}}_{\mathrm{liv}} \cdot \frac{\mathrm{RET}^{\mathrm{vi}}_{\mathrm{liv}}(t)}{V^{\mathrm{vi}}_{\mathrm{liv}}} \\ &\quad + \mathrm{CL}_{\mathrm{act,ef}} \cdot \mathrm{fu}^{\mathrm{c}}_{\mathrm{liv}} \cdot \frac{\mathrm{RET}^{\mathrm{c}}_{\mathrm{liv}}(t)}{V^{\mathrm{c}}_{\mathrm{liv}}} + \mathrm{PS}_{\mathrm{diff}} \cdot \frac{\mathrm{fn}^{\mathrm{c}}_{\mathrm{liv}}}{\mathrm{fn}^{\mathrm{int}}_{\mathrm{liv}}} \cdot \mathrm{fu}^{\mathrm{c}}_{\mathrm{liv}} \cdot \frac{\mathrm{RET}^{\mathrm{c}}_{\mathrm{liv}}(t)}{V^{\mathrm{c}}_{\mathrm{liv}}} \\ &\text{with } K^{\mathrm{vas:vi}}_{\mathrm{liv}} = \frac{\mathrm{BP}}{\mathrm{fuP}} \cdot \left(\frac{V^{\mathrm{vas}}_{\mathrm{liv}}}{V^{\mathrm{vi}}_{\mathrm{liv}}} \cdot \frac{\mathrm{BP}}{\mathrm{fuP}} + \frac{V^{\mathrm{int}}_{\mathrm{liv}}}{V^{\mathrm{vi}}_{\mathrm{liv}}} \cdot \frac{1}{\mathrm{fu}^{\mathrm{int}}_{\mathrm{liv}}}\right)^{-1}, \\ &\quad K^{\mathrm{int,u:vi}}_{\mathrm{liv}} = \mathrm{fu}^{\mathrm{int}}_{\mathrm{liv}} \cdot \left(\frac{V^{\mathrm{vas}}_{\mathrm{liv}}}{V^{\mathrm{vi}}_{\mathrm{liv}}} \cdot \frac{\mathrm{BP}}{\mathrm{fuP}} + \frac{V^{\mathrm{int}}_{\mathrm{liv}}}{V^{\mathrm{vi}}_{\mathrm{liv}}} \cdot \frac{1}{\mathrm{fu}^{\mathrm{int}}_{\mathrm{liv}}}\right)^{-1}\end{aligned} \tag{S10}$$

Retrorsine in liver cellular (superscript c) space:

$$\begin{aligned}\frac{d\mathrm{RET}^{\mathrm{c}}_{\mathrm{liv}}(t)}{dt} &= (\mathrm{CL}_{\mathrm{act,in}} + \mathrm{PS}_{\mathrm{diff}}) \cdot K^{\mathrm{int,u:vi}}_{\mathrm{liv}} \cdot \frac{\mathrm{RET}^{\mathrm{vi}}_{\mathrm{liv}}(t)}{V^{\mathrm{vi}}_{\mathrm{liv}}} - \mathrm{CL}_{\mathrm{act,ef}} \cdot \mathrm{fu}^{\mathrm{c}}_{\mathrm{liv}} \frac{\mathrm{RET}^{\mathrm{c}}_{\mathrm{liv}}(t)}{V^{\mathrm{c}}_{\mathrm{liv}}} \\ &\quad - \mathrm{PS}_{\mathrm{diff}} \frac{\mathrm{fn}^{\mathrm{c}}_{\mathrm{liv}}}{\mathrm{fn}^{\mathrm{int}}_{\mathrm{liv}}} \cdot \mathrm{fu}^{\mathrm{c}}_{\mathrm{liv}} \frac{\mathrm{RET}^{\mathrm{c}}_{\mathrm{liv}}(t)}{V^{\mathrm{c}}_{\mathrm{liv}}} - \mathrm{CL}_{\mathrm{bile}} \cdot \mathrm{fu}^{\mathrm{c}}_{\mathrm{liv}} \frac{\mathrm{RET}^{\mathrm{c}}_{\mathrm{liv}}(t)}{V^{\mathrm{c}}_{\mathrm{liv}}} \\ &\quad - 1 \cdot \frac{V_{\mathrm{max,liv}}}{K_{\mathrm{M,liv}} + \mathrm{fu}^{\mathrm{c}}_{\mathrm{liv}} \frac{\mathrm{RET}^{\mathrm{c}}_{\mathrm{liv}}(t)}{V^{\mathrm{c}}_{\mathrm{liv}}}} \cdot \mathrm{fu}^{\mathrm{c}}_{\mathrm{liv}} \frac{\mathrm{RET}^{\mathrm{c}}_{\mathrm{liv}}(t)}{V^{\mathrm{c}}_{\mathrm{liv}}} \\ &\text{with } 1 = f_{\mathrm{DHR:GSH}} + f_{\mathrm{DHR:DNA}} + f_{\mathrm{DHR:PROT}} + f_{\mathrm{other}}\end{aligned} \tag{S11}$$

Retrorsine in bile:

$$\frac{d\mathrm{RET}_{\mathrm{bile}}(t)}{dt} = \mathrm{CL}_{\mathrm{bile}} \cdot \mathrm{fu}^{\mathrm{c}}_{\mathrm{liv}} \frac{\mathrm{RET}^{\mathrm{c}}_{\mathrm{liv}}(t)}{V^{\mathrm{c}}_{\mathrm{liv}}} \tag{S12}$$

GSH conjugates (DHR:GSH) in liver cellular space:

$$\begin{aligned}\frac{d\mathrm{DHR}:\mathrm{GSH}^{\mathrm{c}}_{\mathrm{liv}}(t)}{dt} = & f_{\mathrm{DHR:GSH}} \frac{V_{\mathrm{max,liv}}}{K_{\mathrm{M,liv}} + \mathrm{fu}^{\mathrm{c}}_{\mathrm{liv}} \frac{\mathrm{RET}^{\mathrm{c}}_{\mathrm{liv}}(t)}{V^{\mathrm{c}}_{\mathrm{liv}}}} \cdot \mathrm{fu}^{\mathrm{c}}_{\mathrm{liv}} \cdot \frac{\mathrm{RET}^{\mathrm{c}}_{\mathrm{liv}}(t)}{V^{\mathrm{c}}_{\mathrm{liv}}} \\ & - \mathrm{e}^{-\lambda_{\mathrm{DHR:GSH}} \cdot t} \cdot \mathrm{DHR}:\mathrm{GSH}^{\mathrm{c}}_{\mathrm{liv}}(t)\end{aligned} \tag{S13}$$

Protein adducts (DHR:PROT) in liver cellular space:

$$\begin{aligned}\frac{d\mathrm{DHR}:\mathrm{PROT}^{\mathrm{c}}_{\mathrm{liv}}(t)}{dt} = & f_{\mathrm{DHR:PROT}} \frac{V_{\mathrm{max,liv}}}{K_{\mathrm{M,liv}} + \mathrm{fu}^{\mathrm{c}}_{\mathrm{liv}} \frac{\mathrm{RET}^{\mathrm{c}}_{\mathrm{liv}}(t)}{V^{\mathrm{c}}_{\mathrm{liv}}}} \cdot \mathrm{fu}^{\mathrm{c}}_{\mathrm{liv}} \frac{\mathrm{RET}^{\mathrm{c}}_{\mathrm{liv}}(t)}{V^{\mathrm{c}}_{\mathrm{liv}}} \\ & - \mathrm{e}^{-\lambda_{\mathrm{DHR:PROT}} \cdot t} \cdot \mathrm{DHR}:\mathrm{PROT}^{\mathrm{c}}_{\mathrm{liv}}(t)\end{aligned} \tag{S14}$$

DNA adducts (DHR:DNA) in liver cellular space:

$$\begin{aligned}\frac{d\mathrm{DHR}:\mathrm{DNA}^{\mathrm{c}}_{\mathrm{liv}}(t)}{dt} = & f_{\mathrm{DHR:DNA}} \frac{V_{\mathrm{max,liv}}}{K_{\mathrm{M,liv}} + \mathrm{fu}^{\mathrm{c}}_{\mathrm{liv}} \frac{\mathrm{RET}^{\mathrm{c}}_{\mathrm{liv}}(t)}{V^{\mathrm{c}}_{\mathrm{liv}}}} \cdot \mathrm{fu}^{\mathrm{c}}_{\mathrm{liv}} \frac{\mathrm{RET}^{\mathrm{c}}_{\mathrm{liv}}(t)}{V^{\mathrm{c}}_{\mathrm{liv}}} \\ & - (\lambda_{2,\mathrm{DHR:DNA}} + \lambda_{1,\mathrm{DHR:DNA}} - \lambda_{2,\mathrm{DHR:DNA}}) \cdot \mathrm{e}^{-k_{\mathrm{DHR:DNA}} \cdot t} \\ & \cdot \mathrm{DHR}:\mathrm{DNA}^{\mathrm{c}}_{\mathrm{liv}}(t)\end{aligned} \tag{S15}$$

Table S1 Final parameters of the PBTK model of retrorsine in mouse and rat. Parameters identified by maximum likelihood estimation based on the training dataset are reported as posterior mode and 95% credible interval (shaded grey). Parameters derived from *in vitro* experiments are shaded blue

Parameter	Unit	Additional description	Mouse	Rat	Source or [95% credible interval]
**Body Weight**†					
bw	kg		0.0250	0.250	Brown et al. (1997)
**Tissue Volumes**†					
V_{blo}	L	Blood	$1.02 \cdot 10^{-3}$	$9.46 \cdot 10^{-3}$	Diehl et al. (2001)
V_{ven}	L	Venous blood	$6.80 \cdot 10^{-4}$	$6.31 \cdot 10^{-3}$	Assumed $2/3$ of V_{blo}
V_{art}	L	Arterial blood	$3.40 \cdot 10^{-4}$	$3.15 \cdot 10^{-3}$	Assumed $1/3$ of V_{blo}
V_{adi}	L	Adipose	$1.91 \cdot 10^{-3}$	0.0289	Brown et al. (1997)
V_{bon}	L	Bone	$2.06 \cdot 10^{-3}$	0.0140	Brown et al. (1997)
V_{bra}	L	Brain	$4.13 \cdot 10^{-4}$	$1.43 \cdot 10^{-3}$	Brown et al. (1997)
V_{gut}	L	Gut tissue	$1.06 \cdot 10^{-3}$	$6.75 \cdot 10^{-3}$	Brown et al. (1997)
V_{hea}	L	Heart	$1.25 \cdot 10^{-4}$	$8.25 \cdot 10^{-4}$	Brown et al. (1997)
V_{kid}	L	Kidneys	$4.18 \cdot 10^{-4}$	$1.83 \cdot 10^{-3}$	Brown et al. (1997)
V_{liv}	L	Liver volume	$1.37 \cdot 10^{-3}$	$9.15 \cdot 10^{-3}$	Brown et al. (1997)
V_{liv}^{c}	L	Liver cellular space	$9.91 \cdot 10^{-4}$	$6.61 \cdot 10^{-3}$	Kawai et al. (1994)
V_{liv}^{vi}	L	Liver vascular/interstitial space	$3.82 \cdot 10^{-4}$	$2.54 \cdot 10^{-3}$	Kawai et al. (1994)
V_{lun}	L	Lungs	$1.83 \cdot 10^{-4}$	$1.25 \cdot 10^{-3}$	Brown et al. (1997)
V_{mus}	L	Muscle	$9.60 \cdot 10^{-3}$	0.101	Brown et al. (1997)
V_{ski}	L	Skin	$4.13 \cdot 10^{-3}$	0.0476	Brown et al. (1997)
V_{spl}	L	Spleen	$8.75 \cdot 10^{-5}$	$5.00 \cdot 10^{-4}$	Brown et al. (1997)
**Tissue Blood Flows**†					
Q_{adi}	L/h	Adipose	0.0726	0.349	Brown et al. (1997); El-Masri and Portier (1998)
Q_{bon}	L/h	Bone	0.114	0.608	Brown et al. (1997); El-Masri and Portier (1998)
Q_{bra}	L/h	Brain	0.0342	0.0997	Brown et al. (1997); El-Masri and Portier (1998)
Q_{gut}	L/h	Gut tissue	0.146	0.653	Brown et al. (1997); El-Masri and Portier (1998)
Q_{hea}	L/h	Heart	0.0685	0.244	Brown et al. (1997)
Q_{kid}	L/h	Kidneys	0.0944	0.703	Brown et al. (1997)
Q_{liv}	L/h	Liver	0.168	0.867	Brown et al. (1997)
Q_{mus}	L/h	Muscle	0.165	1.39	Brown et al. (1997)
Q_{ski}	L/h	Skin	0.0602	0.289	Brown et al. (1997)
Q_{spl}	L/h	Spleen	0.0104	0.0997	El-Masri and Portier (1998); Poulin and Theil (2002)
**Cardiac Output**†					
Q_c	L/h		0.777	4.55	Brown et al. (1997)
**Glomerular Filtration Rate**†					
GFR	L/h		0.0120	0.120	Benjamin et al. (2015)
**Hematocrit**					
hct	fraction	Fraction of red blood cells in blood	Both species: 0.400		Baskurt et al. (2007)
**Physico-/ Biochemical Properties**					
subclass	-	Neutral/ acid/ weak base/ strong base	Both species: weak base		
pK_a	-	Acid strength	Both species: 6.86		Predicted using the web application SPARC (ARChem 2019)
log P	-	Lipophilicity	Both species: -1.26		Haas et al. (2019)
BP	-	Blood-to-plasma ratio	Both species: 1.08		Assumed equal to human BP, Haas et al. (2019)
fuP	fraction	Fraction unbound in blood plasma	Both species: 0.600		Assumed equal to human fuP, Haas et al. (2019)
fu_{liv}^{int}	fraction	Fraction unbound in liver interstitium	Both species: 0.600		Assumed equal to fuP
fu_{liv}^{c}	fraction	Fraction unbound in liver cell	Both species: 0.600		Assumed equal to fuP
fu_{gut}	fraction	Fraction unbound in gut tissue	Both species: 0.600		Assumed equal to fuP
fn_{liv}^{int}	fraction	Fraction neutral in liver interstitium	Both species: 0.776		Henderson-Hasselbalch equation
fn_{liv}^{c}	fraction	Fraction neutral in liver cell	Both species: 0.701		Henderson-Hasselbalch equation
**Tissue-to-Plasma Partition Coefficients**†					
K_{adi}	-	Adipose	Both species: 0.184		Predicted as in Rodgers and Rowland (2006)
K_{bon}	-	Bone	Both species: 0.602		Predicted as in Rodgers and Rowland (2006)
K_{bra}	-	Brain	Both species: 0.997		Predicted as in Rodgers and Rowland (2006)
K_{gut}	-	Gut tissue	Both species: 1.01		Predicted as in Rodgers and Rowland (2006)
K_{hea}	-	Heart	Both species: 0.833		Predicted as in Rodgers and Rowland (2006)
K_{kid}	-	Kidneys	Both species: 0.936		Predicted as in Rodgers and Rowland (2006)
$K_{liv}^{vas:vi}$	-	Liver vascular-to-lumped compartment partition coeffcient	Both species: 1.05		Predicted as in Schweinoch (2014), Eq.S10
$K_{liv}^{int,u:vi}$	-	Liver unbound interstitial-to-lumped compartment partition coeffcient	Both species: 0.348		Predicted as in Schweinoch (2014), Eq.S10

Continued on next page

Table S1 Continued from previous page

Parameter	Unit	Additional description	Mouse	Rat	Source or [95% credible interval]
K_{lun}	-	Lungs	Both species: 0.874		Predicted as in Rodgers and Rowland (2006)
K_{mus}	-	Muscle	Both species: 0.986		Predicted as in Rodgers and Rowland (2006)
K_{ski}	-	Skin	Both species: 0.946		Predicted as in Rodgers and Rowland (2006)
K_{spl}	-	Spleen	Both species: 0.829		Predicted as in Rodgers and Rowland (2006)
**Liver Transport**					
$\mathrm{PS}_{\mathrm{diff}}$	mL/min/g liver	Passive influx diffusion flow rate	0.190	0.394	Medium loss assay
$\mathrm{CL}_{\mathrm{act,in}}$	mL/min/g liver	Active uptake clearance	0.516	0.887	Medium loss assay
$\mathrm{CL}_{\mathrm{act,ef}}$	mL/min/g liver	Active efflux clearance	Both species: 0		Assumed negligible
$\mathrm{CL}_{\mathrm{bile}}$	mL/min/g liver	Biliary clearance	0	0.101	Assumed negligible (mouse) [0.0878, 0.118] (rat)
**Liver Metabolism**					
$K_{\mathrm{M,liv}}$	μM	Concentration at half-maximal reaction velocity	55.4	44.3	Liver microsomal assay
$V_{\mathrm{max,liv}}$	nmol/min/g liver	Maximum reaction velocity	27.6	91.7	Liver microsomal assay
**Glutathione conjugates (DHR:GSH)**					
$f_{\mathrm{DHR:GSH}}$	fraction	Fraction metabolized to DHR:GSH	Both species: $5.11 \cdot 10^{-3}$		$[3.73 \cdot 10^{-3}, 7.76 \cdot 10^{-3}]$
$\lambda_{\mathrm{DHR:GSH}}$	1/h	Depletion rate constant of DHR:GSH	Both species: 4.94		[3.38, 7.87]
**Protein adducts (DHR:PROT)**					
$f_{\mathrm{DHR:PROT}}$	fraction	Fraction metabolized to DHR:PROT	Both species: $2.61 \cdot 10^{-3}$		$[1.86 \cdot 10^{-3}, 3.72 \cdot 10^{-3}]$
$\lambda_{\mathrm{DHR:PROT}}$	1/h	Depletion rate constant of DHR:PROT	Both species: 0.0847		[0.0481, 0.162]
**DNA adducts (DHR:DNA)**					
$f_{\mathrm{DHR:DNA}}$	fraction	Fraction metabolized to DHR:DNA	Both species: $1.48 \cdot 10^{-6}$		$[1.25 \cdot 10^{-6}, 2.82 \cdot 10^{-6}]$
$\lambda_{1,\mathrm{DHR:DNA}}$	1/h	Depletion rate constant of DHR:DNA (1st phase)	Both species: 0.0926		[0.0413, 0.611]
$\lambda_{2,\mathrm{DHR:DNA}}$	1/h	Depletion rate constant of DHR:DNA (2nd phase)	Both species: $2.91 \cdot 10^{-3}$		$[1.81 \cdot 10^{-3}, 4.42 \cdot 10^{-3}]$
$k_{\mathrm{DHR:DNA}}$	1/h	Transition rate constant of DHR:DNA	Both species: 0.106		[0.0576, 0.260]
**Other Metabolites**					
f_{other}	fraction	Fraction metabolized to other retrorsine metabolites, e.g. N-oxide, products of hydrolysis	Both species: 0.992		Equals 1-($f_{\mathrm{DHR:GSH}}$+$f_{\mathrm{DHR:DNA}}$ +$f_{\mathrm{DHR:PROT}}$)
**Intestinal Metabolism**					
$K_{\mathrm{M,gut}}$	μM	Concentration at half-maximal reaction velocity	55.4	44.3	Assumed equal to $\mathrm{K}_{\mathrm{M,liv}}$
$V_{\mathrm{max,gut}}$	nmol/min/g liver	Maximum reaction velocity	2.76	9.17	Assumed $\frac{1}{10} \cdot V_{\mathrm{max,liv}}$
**Intestinal Absorption**					
F_{a}	fraction	Intestinal fraction absorbed	Both species: 0.783		Caco-2 permeability assay
k_{a}	1/h	Intestinal absorption rate constant	Both species: 0.910		[0.581, 3.51]
**Peritoneal Absorption**					
k_{per}	1/h	Peritoneal absorption rate constant	Both species: 166		Fixed; $t_{1/2} = 15$ sec

† Physiological parameters and prediction of tissue-to-plasma partition coefficients were based on the MATLAB-based pharmacometric modeling framework developed by Hartung and Huisinga (2019)

Table S2 Kinetic *in vivo* studies with retrorsine

Data	Tissue	Species	Adm[a]	Dose (mg/kg bw)	Data reporting	Function
**White (1977)**						
RET	bile	rat	i.v.	40	summary	model training
**Chu and Segall (1991)**						
RET	urine	mouse<br>rat	i.p.	25	summary	model training
**Yang et al. (2017)**						
RET	plasma	mouse	i.p.	70	individual (personal correspondance)	model training
DHR:GSH	liver					
DHR:PROT	liver					
**Zhu et al. (2017)**						
DHR:DNA	liver	mouse	p.o.	10, 20, 40, 60	summary	model training<br>model evaluation
**Yang et al. (2018)**						
RET	plasma<br>liver	mouse	i.p.	50	summary	model evaluation
DHR:GSH	liver					
DHR:PROT	liver					
GSH	liver					
**Li et al. (2022)**						
RET	serum [b]	mouse	p.o.<br>i.v.	20<br>5	summary<br>summary	model training<br>model evaluation

DHR:GSH dehydroretronecine glutathione conjugate; DHR:PROT dehydroretronecine protein adduct; DHR:DNA dehydroretronecine DNA adduct; GSH glutathione; RET retrorsine

[a] Administration (Adm) routes: intraperitoneal (i.p.), intravenous (i.v.) or per oral (p.o.)

[b] Serum was treated as plasma in the PBTK model

Pre-processing of *in vivo* kinetic data

Kinetic data from *in vivo studies* were standardized into one common base unit, amount of substance (nmol):
Retrorsine (RET) data were either reported as % of dose in urine and bile or as µg/mL in plasma and liver. The conversion of RET data into amount of substance (nmol) is shown in Eqs. S16-S19.
Glutathione conjugate (DHR:GSH) and protein adduct (DHR:PROT) data were reported as $A_{\text{Analyte}}/A_{\text{IS}}/\text{mg}$ protein expressing the ratio of peak areas of analyte and internal standard (IS) in LC-MS/MS per mg protein of supernatant from liver homogenates. It was assumed that the mass spectrometric response of analyte and internal standard are similar. However, due to lack of mass spectrometric standards for quantification of DHR:GSH and DHR:PROT, data transformed to amount of substance (nmol) have to be regarded as relative quantities (Eqs. S20-S21). In the experimental protocolYang et al. (2017, 2018) reported that mouse livers were homogenized and the resulting supernatants were used for mass spectrometric analysis. To account for the difference of protein amount in supernatants versus total liver homogenates, DHR:GSH and DHR:PROT data were multiplied by the protein ratio R_{prot} of supernatant versus homogenate (Eqs. S20-S21), which was provided by personal correspondance with X. Yang (Yang et al. 2017).

DNA adduct (DHR:DNA) data were transformed from adducts/10^8 nucleotides to adducts/liver in nmol using scaling factors (Eq. S22). SF_{genome} describes the number of base pairs/ haploid cell that is identical to the number of nucleotide pairs/ haploid cell. By multiplication with SF_{genome} DHR:DNA were tranformed to adducts/ cell. We accounted for individual nucleotides by multiplication with factor 2, as well as for a diploid cell also using factor 2. The hepatocellularity SF_{liv} scales adducts/cell to adducts/g liver. The Avogadro constant N_A relates the number of hepatic adduct molecules to amount of substance.

Data from Chu and Segall (1991) and White (1977):

$$\mathrm{RET_{tis}\ (nmol)} = \frac{\mathrm{RET_{tis}\ (\%\ of\ dose)}}{100} \cdot \frac{D \cdot \mathrm{bw}}{\mathrm{M_{RET}}} \cdot 10^6 \qquad \mathrm{tis} = \mathrm{uri}, \mathrm{bile} \tag{S16}$$

where:

bw	Body weight (kg):	0.0250 (mouse)	(Brown et al. 1997)
		0.250 (rat)	
D	Dose (mg/kg bw):	Study-specific	
M_{RET}	RET molar mass (g/mol):	351.4	(NCBI 2021c)
tis	Abbreviation for tissue		
uri	Abbreviation for urine		

Data from Li et al. (2022):

$$\mathrm{RET_{pla}\ (nmol)} = \mathrm{RET_{pla}}\ (\mu\mathrm{g/mL}) \cdot \frac{V_{\mathrm{pla}}}{\mathrm{M_{RET}}} \cdot 10^6 \tag{S17}$$

where:

M_{RET}	RET molar mass (g/mol):	351.4	(NCBI 2021c)
V_{pla}	Blood plasma volume (L):	$V_{blo} \cdot (1 - \mathrm{hct}) = 6.12 \cdot 10^{-4}$	(Hartung and Huisinga 2019)
V_{blo}	Blood volume (L):	$1.02 \cdot 10^{-3}$	(Diehl et al. 2001)
hct	Hematocrit (-):	0.400	(Baskurt et al. 2007)

Data from Yang et al. (2017) and Yang et al. (2018):

$$\mathrm{RET_{liv}\ (nmol)} = \mathrm{RET_{liv}}\ (\mu\mathrm{g/mL}) \cdot \frac{V_{\mathrm{liv}}}{\mathrm{M_{RET}}} \cdot \mathrm{DF_{liv}} \cdot 10^6 \tag{S18}$$

$$\mathrm{RET_{liv}\ (nmol)} = \mathrm{RET_{pla}}\ (\mu\mathrm{g/mL}) \cdot \frac{V_{\mathrm{pla}}}{\mathrm{M_{RET}}} \cdot 10^6 \tag{S19}$$

where:

DF_{liv}	Liver dilution factor (-):	$\frac{\text{2 g buffer + 0.2 g liver sample}}{\text{0.2 g liver sample}} = 11$	(Derived from the experimental protocol in Yang et al. (2018))
liv	Abbreviation for liver		
M_{RET}	RET molar mass (g/mol):	351.4	(NCBI 2021c)
pla	Abbreviation for blood plasma		
tis	Abbreviation for tissue		
V_{liv}	Liver volume (L):	$1.37 \cdot 10^{-3}$	(Brown et al. 1997)
V_{pla}	Blood plasma volume (L):	$V_{blo} \cdot (1 - \text{hct}) = 6.12 \cdot 10^{-4}$	(Hartung and Huisinga 2019)
V_{blo}	Blood volume (L):	$1.02 \cdot 10^{-3}$	(Diehl et al. 2001)
hct	Hematocrit (-):	0.400	(Baskurt et al. 2007)

$$\begin{aligned}\text{DHR : GSH}_{\text{liv}} \text{ (nmol, relative)} = {} & \text{DHR : GSH}_{\text{liv}} \ (A_{\text{Analyte}}/A_{\text{IS}}/\text{mg protein}) \\ & \cdot C_{\text{prot,liv}} \cdot W_{\text{liv}} \cdot \frac{W_{\text{IS,DHR:GSH}}}{M_{\text{IS,DHR:GSH}}} \cdot R_{\text{prot}} \cdot 10^9 \end{aligned} \tag{S20}$$

$$\begin{aligned}\text{DHR : PROT}_{\text{liv}} \text{ (nmol, relative)} = {} & \text{DHR : PROT}_{\text{liv}} \ (A_{\text{Analyte}}/A_{\text{IS}}/\text{mg protein}) \\ & \cdot C_{\text{prot,liv}} \cdot W_{\text{liv}} \cdot \frac{W_{\text{IS,DHR:PROT}}}{M_{\text{IS,DHR:PROT}}} \cdot R_{\text{prot}} \cdot 10^9 \end{aligned} \tag{S21}$$

where:

$A_{Analyte}$	Amount of analyte in mass spectrometry		
A_{IS}	Amount of internal standard in mass spectrometry		
$C_{prot,liv}$	Liver protein conc. (mg protein/mg liver):	0.238	(Verma and Chakraborty 2008)
IS	Abbreviation for internal standard		
liv	Abbreviation for liver		
$M_{IS,DHR:GSH}$	Molar mass of S-hexylglutathione (g/mol):	392	(NCBI 2021d)
$M_{IS,DHR:PROT}$	Molar mass of diazepam (g/mol):	285	(NCBI 2021b)
R_{prot}	Protein ratio (-):	0.787	(Personal correspondance with X. Yang (Yang et al. 2017))
$W_{IS,DHR:GSH}$	Mass of S-hexylglutathione (g):	$1.25 \cdot 10^{-8}$	(Yang et al. 2017)
		$1.6 \cdot 10^{-8}$	(Yang et al. 2018)
$W_{IS,DHR:PROT}$	Mass of diazepam (g):	$1.25 \cdot 10^{-8}$	(Yang et al. 2017)
W_{liv}	Liver weight (mg):	1370	(Brown et al. 1997)

Data from Zhu et al. (2017):

$$\begin{aligned}\text{DHR : DNA}_{\text{liv}} \text{ (nmol)} = {} & \text{DHR : DNA}_{\text{liv}} \ (\text{adducts}/10^8 \text{ nucleotides}) \\ & \cdot \text{SF}_{\text{genome}} \cdot 2 \cdot 2 \cdot 10^8 \cdot \text{SF}_{\text{liv}} \cdot W_{\text{liv}} \cdot \frac{1}{N_{\text{A}}} \cdot 10^9 \end{aligned} \tag{S22}$$

where:

N_A	Avogadro constant (1/mol):	$6.02 \cdot 10^{23}$	(IUPAC 1997)
SF_{genome}	Scaling factor (bp/haploid cell):	$2.73 \cdot 10^{9}$	(NCBI 2021a)
SF_{liv}	Hepatocellularity (cells/g liver):	$1.28 \cdot 10^{8}$	(Ring et al. 2011)
W_{liv}	Liver weight (g):	1.37	(Brown et al. 1997)

Table S3 Characteristics of mouse and rat liver microsomal preparations as provided by the manufacturer

Characteristic	Unit	Mouse	Rat
Strain	-	CD-1	Sprague Dawley
Sex	-	male	male
Age	weeks	11	8-10
No. of pooled livers	-	210	132
Protein concentration	mg/mL	20	20
Cytochrome P450 content	pmol/mg protein	740	660
CYP1A activity	pmol/mg/min	180	290
CYP2C activity	pmol/mg/min	not provided	5400
CYP3A activity	pmol/mg/min	4200	3900

Chemicals and Biological Materials

Retrorsine was purchased from AppliChem (Darmstadt, Germany) and from Phytoplan (Heidelberg, Germany). William's Medium E and supplements were puchased from PAN-Biotech (Aidenbach, Germany). Methanol and water (LC–MS grade) were obtained from Merck KGA (Darmstadt, Germany). All other chemicals and co-factors for liver microsomal assays were purchased from Carl Roth (Karlsruhe, Germany) or Sigma-Aldrich (Steinheim, Germany). Liver microsomes of CD-1 mice (11 weeks old) and Sprague-Dawley rats (eight to 10 weeks old) were obtained by Corning (Woburn, MA, USA). Characteristics of microsomal preparations are summarized in Table S3. Male CD-1 mice (eight weeks old) for isolation of primary hepatocytes were purchased from Janvier Labs (Le Genest-Saint-Isle, France). Male Sprague-Dawley rats (weighing 180 to 200 g) for isolation of primary hepatocytes were purchased from Charles River Laboratories (Wilmignton, MA, USA). The human colon adenocarcinoma cell line Caco-2 was obtained from the European Collection of Cell Cultures (ECACC, Porton Down, UK).

Isolation and Culture of Primary Hepatocytes

Primary hepatocytes were isolated from male CD-1 mice and male Sprague-Dawley rats by the two-step collagenase perfusion method. A detailed protocol of isolation including materials is given in Godoy et al. (2013) according to the protocol 'Isolation of primary rat and mouse hepatocytes' in Appendix I (see pages 1471-1475).
Isolated cells were plated as a monolayer on 12-well plates onto dried collagen I at a density of $4.5 \cdot 10^5$ cells/mL in pre-warmed culture medium. The culture medium consisted of William's E medium supplemented with 100 U/mL penicillin/0.1 mg/mL streptomycin, 50 µg/mL gentamycin, 100 nM dexamethasone in EtOH, 20 mM L-glutamine, 2 ng/mL insulin and 10% fetal calf serum. Hepatocytes were incubated at 37°C under an atmosphere of 5% CO_2 and left for 3 h to attach. Then, cells were washed three times with William's E medium before the start of the medium loss assay and the cytotoxicity assay. A detailed protocol of the collagen monolayer preparation procedure including materials is given in Godoy et al. (2013) according to the protocol 'Collagen monolayer protocol' in Appendix II (see page 1477).

Medium Loss Assay

Three hours after seeding, cultured hepatocytes were used for the medium loss assay. At the start of the experiment, cells were treated with 0.7 μM retrorsine diluted in serum-free incubation medium either at 37°C or at 4°C. Time-dependent retrorsine depletion was measured and therefore medium samples were taken at 0, 2.5, 5, 10, 30 and 60 min. Samples were stored at -20°C until they were diluted in 5% methanol and analysed for retrorsine by LC-MS/MS as reported in Geburek et al. (2020). Primary hepatocytes of three (rat) or two (mouse) animals were used as biological replicates and for each animal three technical replicates were examined.
Measured retrorsine concentrations were normalized to the inital retrorsine concentration RET_0. Retrorsine depletion-time profiles were described by a monoexponential decay model, where the parameter λ_T (1/h) represents the rate constant of retrorsine loss in medium at the respective temperature T:

$$\frac{\mathrm{RET}(t)}{\mathrm{RET}_0} = e^{-\lambda_\mathrm{T}\cdot t}$$
$$\mathrm{T}(^\circ\mathrm{C}) = 4,37 \tag{S23}$$

A two-compartment model (Fig. S1) was used to derive the passive influx diffusion flow rate into the cells $\mathrm{PS}_{\mathrm{diff,in}\,\textit{in vitro}}$ and the active uptake clearance $\mathrm{CL}_{\mathrm{act,in}\,\textit{in vitro}}$ (both L/h/10^6 cells). $\mathrm{PS}_{\mathrm{diff,in}\,\textit{in vitro}}$ and $\mathrm{CL}_{\mathrm{act,in}\,\textit{in vitro}}$ were approximated under the assumption that passive diffusion out of the cells is negligible during the first monoexponential phase (Eqs. S24-S25). *In vitro*-to-*in vivo* extrapolation of both parameters was performed by multiplication with hepatocellularity SF_liv and fraction unbound *in vitro* $\mathrm{fu}_{\textit{in vitro}}$ yielding *in vivo* $\mathrm{PS}_\mathrm{diff,in}$ and $\mathrm{CL}_\mathrm{act,in}$ (both L/h/g liver) (Eqs. S26 -S27).

$$\mathrm{PS}_{\mathrm{diff,in}\,\textit{in vitro}} = \lambda_{4^\circ\mathrm{C}} \cdot \frac{1}{C_\mathrm{cell}} \tag{S24}$$
$$\mathrm{CL}_{\mathrm{act,in}\,\textit{in vitro}} = \lambda_{37^\circ\mathrm{C}} \cdot \frac{1}{C_\mathrm{cell}} - \mathrm{PS}_{\mathrm{diff,in}\,\textit{in vitro}} \tag{S25}$$
$$\mathrm{PS}_\mathrm{diff,in} = \mathrm{PS}_{\mathrm{diff,in}\,\textit{in vitro}} \cdot \mathrm{SF}_\mathrm{liv} \cdot \mathrm{fu}_{\textit{in vitro}} \tag{S26}$$
$$\mathrm{CL}_\mathrm{act,in} = \mathrm{CL}_{\mathrm{act,in}\,\textit{in vitro}} \cdot \mathrm{SF}_\mathrm{liv} \cdot \mathrm{fu}_{\textit{in vitro}} \tag{S27}$$

where:

C_cell	Cell density (cells/mL):	$4.50 \cdot 10^5$	(Experimental)
$\mathrm{fu}_{\textit{in vitro}}$	Fraction unbound *in vitro*	1.00	(Assumption)
SF_liv	Hepatocellularity (cells/g liver):	$128 \cdot 10^6$ (mouse, rat)	(Ring et al. 2011)

$\mathrm{fu}_{\textit{in vitro}}$ was assumed 1, which was supported by the predicted value of 0.994 (Eq. 16 in Austin et al. (2005)). The predicted low protein binding is in line with the low lipophilicity of retrorsine (log P=-1.26).

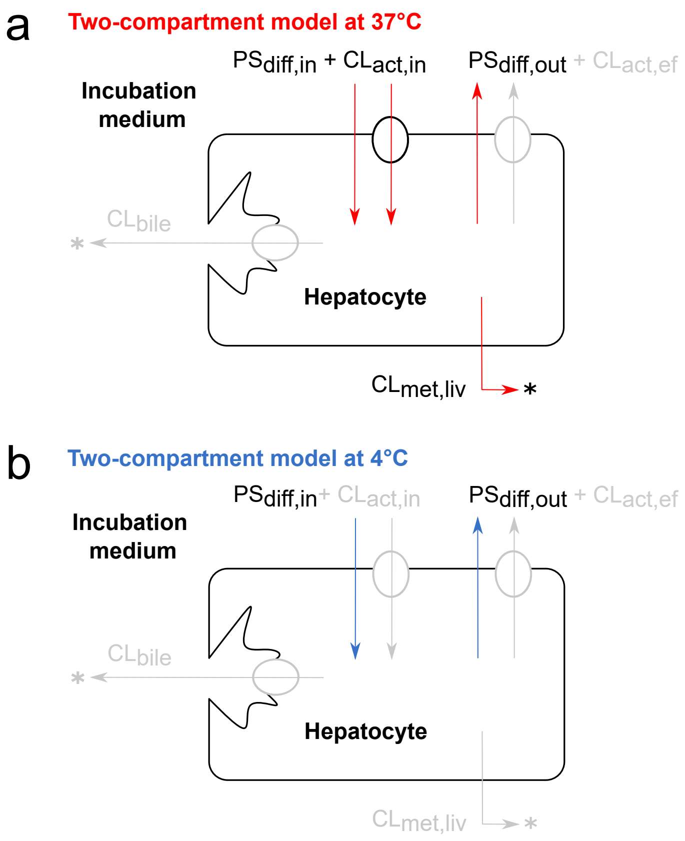

Fig. S1 Two-compartment model characterizing the uptake and clearance processes involved in the medium loss assay based on Schweinoch (2014). The external compartment represents the incubation medium surrounding the hepatocytes, while the internal compartment represents the hepatocytes' intracellular space. It was assumed that in the medium loss assay most compound exporting proteins are not active in unpolarized monolayers of hepatocytes (absence of biliary clearance CL_{bile} and active efflux $CL_{act,ef}$). At a physiological temperature of 37°C active influx $CL_{act,in}$, metabolism $CL_{met,liv}$ and passive diffusion $PS_{diff,in}$, $PS_{diff,out}$ were involved in compound clearance **(a)**. At a non-physiological temperature of 4°C active influx and metabolism were regarded as negligible **(b)**. Note: $PS_{diff,in}$ and $CL_{act,in}$ (Eqs. S24-S27) were approximated under the assumption that passive diffusion out of the cells $PS_{diff,out}$ is negligible during the first monoexponential decay phase

Liver Microsomal Assay

Kinetics of retrorsine hepatic metabolism were determined by measuring retrorsine depletion in the presence of mouse and rat liver microsomes. Incubation mixtures were prepared on ice. Microsomal preparations were diluted in 50 mM Tris-HCl buffer (pH 7.5) to yield a protein concentration of 1 mg protein/mL. For simulation of phase I metabolism 33 mM potassium chloride, 8 mM magnesium chloride, 1 mM nicotinamide adenine dinucleotide phosphate (NADPH), 5 mM glucose-6-phosphate and 0.5 U/mL glucose-6-phosphate dehydrogenase were added. 2 mM glutathione were included to fasciliate formation of phase II glutathione conjugates. Mixtures were incubated at 37°C and 400 rpm with 1, 15, 50 and 200 µM of retrorsine. Reactions were stopped at 8, 15, 20, 30, 40, 50 and 60 min by addition of ice-cold methanol containing 1% ammonium formate. All experiments were performed in duplicate. Samples were vortexted and stored at -80°C. Thawed samples were centrifuged at 14,000xg at 4°C to precipitate salts and proteins. Supernatants were diluted in 5% methanol and were analysed for retrorsinne by LC-MS/MS as previously reported in Geburek et al. (2020).
Measured retrorsine concentrations were normalized to the initial retrorsine concentration RET_0. The following end-product inhibition model was developed to describe time-dependent retrorsine depletion:

$$\mathrm{RET} \xrightarrow{k} P \text{ with } P \text{ inhibiting } k \tag{S28}$$

$$\frac{d\mathrm{RET}(t)}{dt} = -k(P)\cdot \mathrm{RET}(t) \tag{S29}$$

$$\frac{dP(t)}{dt} = k(P)\cdot \mathrm{RET}(t) \tag{S30}$$

$$k(P) = k_0(\mathrm{RET}_0)\cdot\left(1-\frac{P(t)}{P(t)+\mathrm{IC}_{50}}\right) \tag{S31}$$

$$\text{with } k_0(\mathrm{RET}_0) = \frac{V_{\mathrm{max,liv},\mathit{in\,vitro}}}{K_{\mathrm{M,liv}}}\cdot\left(1-\frac{\mathrm{RET}_0}{\mathrm{RET}_0+K_{\mathrm{M,liv}}}\right)$$

In the end-product inhibition model, we assumed that RET (μM) is converted to a mixture of products P with reaction rate constant k (1/h) (Eqs. S28-S30). Highly reactive dehydroretrorsine (DHR), one of RET's reaction products, was considered to unspecifically bind to the active center of microsomal enzymes thereby inhibiting their activity over time. The irreversible inhibition of CYP3A4 by resulting from metabolic activation of RET has been demonstrated *in vitro* by Dai et al. (2010). As a consequence k is inhibited in dependency of the product concentration. Half-maximal inhibition of k is achieved at the product concentration IC_{50} (μM) (Eq. S31). k_0 represents the initial reaction rate constant where inhibition is still absent ($t = 0$). Its dependency on RET_0 was described by a Michaelis-Menten-like relationship (Obach and Reed-Hagen 2002) with maximum reaction velocity $V_{\mathrm{max,liv},\mathit{in\,vitro}}$ (μM/min) and RET concentration at half-maximal reaction velocity K_M (μM). *In vitro*-to-*in vivo* extrapolation of $V_{\mathrm{max,liv},\mathit{in\,vitro}}$ yielded $V_{\mathrm{max,liv}}$ (nmol/min/g liver):

$$V_{\mathrm{max,liv}} = V_{\mathrm{max,liv},\mathit{in\,vitro}}\cdot\frac{1}{\rho_{\mathrm{prot}}}\cdot \mathrm{SF}_{\mathrm{microsomes}}\cdot \mathrm{fu}_{\mathit{in\,vitro}} \tag{S32}$$

where:

ρ_{prot}	Protein concentration (mg protein/mL):	1.00	(Experimental)
$\text{fu}_{\textit{in vitro}}$	Fraction unbound *in vitro*	1.00	(Assumption)
$\text{SF}_{\text{microsomes}}$	Scaling factor (mg protein/g liver):	47.0 (mouse,rat)	(Ring et al. 2011)

$\text{fu}_{\textit{in vitro}}$ was assumed 1, which was supported by the predicted value of 0.998 (Eq. for bases in Turner et al. (2007)). The predicted low protein binding is in line with the low lipophilicity of retrorsine (log P=-1.26).

Caco-2 Permeability Assay

Incubation of Caco-2 cells and transport experiments were performed as previously described in (Hessel et al. 2014). Briefly, Caco-2 cells were seeded on Transwell^{TM} inserts (1.12 cm^2 growth area, 0.4 μm pore size, polycarbonate membrane; Corning B.V. Life Sciences, Amsterdam, The Netherlands) at a density of $6 \cdot 10^5$ cells per insert and cultivated for 21 days. Before and after transport experiments the integrity of the cell monolayer was routinely checked by measuring the transepithelial electrical resistance. At the start of the experiment, 1 μM of retrorsine was placed either on the apical (A) or on the basolateral (BL) side. Medium samples (50 μL) from both sides were collected at 4, 8 and 24 h. All experiments were performed in triplicate. Samples were stored at -20°C until they were analyzed for retrorsine by LC-MS/MS using the workflow reported in (Hessel et al. 2014).
The efflux ratio (ER) of retrorsine was calculated based on apparent permeability (P_{app}, cm/s) values of A→BL and BL→A, where C_{acceptor} (ng/mL) is the concentration in the acceptor compartment over time t (s), V_{acceptor} (mL) is the volume of the acceptor compartment, $C_{0,\text{donor}}$ (ng/mL) is the initial concentration in the donor comparment and A (cm^2) is the Transwell^{TM} growth area. Data were reported as mean ± standard deviation.

$$\text{P}_{\text{app,donor}\rightarrow\text{acceptor}} = \frac{C_{\text{acceptor}} \cdot V_{\text{acceptor}}}{C_{0,\text{donor}} \cdot t \cdot A} \tag{S33}$$

$$\text{ER} = \frac{\text{P}_{\text{app,BL}\rightarrow\text{A}}}{\text{P}_{\text{app,A}\rightarrow\text{BL}}} \tag{S34}$$

The *in vivo* intestinal absorption F_{a} (fraction) was predicted making use of the correlation between Caco-2 permeability and human intestinal absorption, which was reported for a set of 93 diverse compounds by Skolnik et al. (2010) (Eq. S35). We assumed that human F_{a} is equal to that of rat and mouse. This assumption was made based on an *in vivo* study reporting a similar extent of intestinal absorption in rats compared to humans (Zhao et al. 2003).

$$F_{\text{a}} = \frac{0.01 + (1 - 0.01)}{1 + \exp\left(\frac{-5.74 - \log\left(P_{\text{app},A\rightarrow BL}\right)}{0.39}\right)} \tag{S35}$$

where:

$P_{\text{app,A}\rightarrow\text{BL}}$	Caco-2 permeability A → BL (cm/s):	Eq. S33

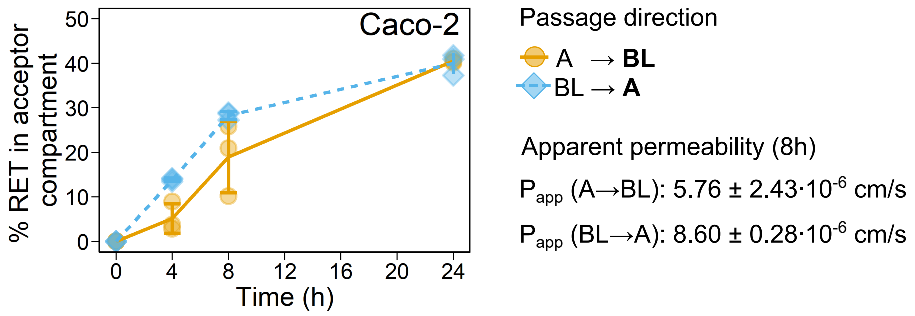

Fig. S2 Time-dependent passage of retrorsine (RET) through the Caco-2 monolayer in the Transwell™ system used as a model for the small intestinal epithelium. Cells were exposed to 1 μM of RET either on the apical (A) or on the basolateral (BL) side. RET levels in the respective acceptor compartment are given as % of the initial RET concentration in the donor compartment of n=3 independent experiments (circles) with mean (solid line) ± standard deviation (error bars)

Cytotoxicity Assay

The cytotoxicity assay was performed using the CellTiter-Blue® (CTB) assay as described in (Gu et al. 2018) according to the SOP in Supplement 3A. Three hours after seeding, cultured hepatocytes were incubated with concentrations of retrorsine ranging between 0 and 250 µM diluted in serum-free incubation medium for a time span of 48 hours. Cells incubated with 10% DMSO were used as a positive control, while cells incubated with medium containing 1.25% acetonitrile were used as solvent control. After incubation, the medium was removed and cells were washed with phosphate-buffered saline. 100 μL serum-free medium containing 20% CTB reagent were added to each well. Supernatants were transferred to black polystyrene 96-well plates and fluorescence was read out with the Tecan Infinite M200 Pro plate reader. Primary mouse and rat hepatocytes of three animals were used as three biological replicates and for each animal three technical replicates were assessed.

Read-outs were corrected for background fluorescence and were normalized animal-wise to fluorescence of the solvent control (representing 100% cell viability at 0 µM retrorsine). The concentration-response curve was described by the sigmoidal inhibition model (Eq. S36), where IC_{50} (μM) is the retrorsine concentration (RET) at half-maximal cell viability.

$$\text{Cell viability} = \frac{100}{1+10^{(\text{RET}-\log \text{IC}_{50})}} \quad \text{(S36)}$$

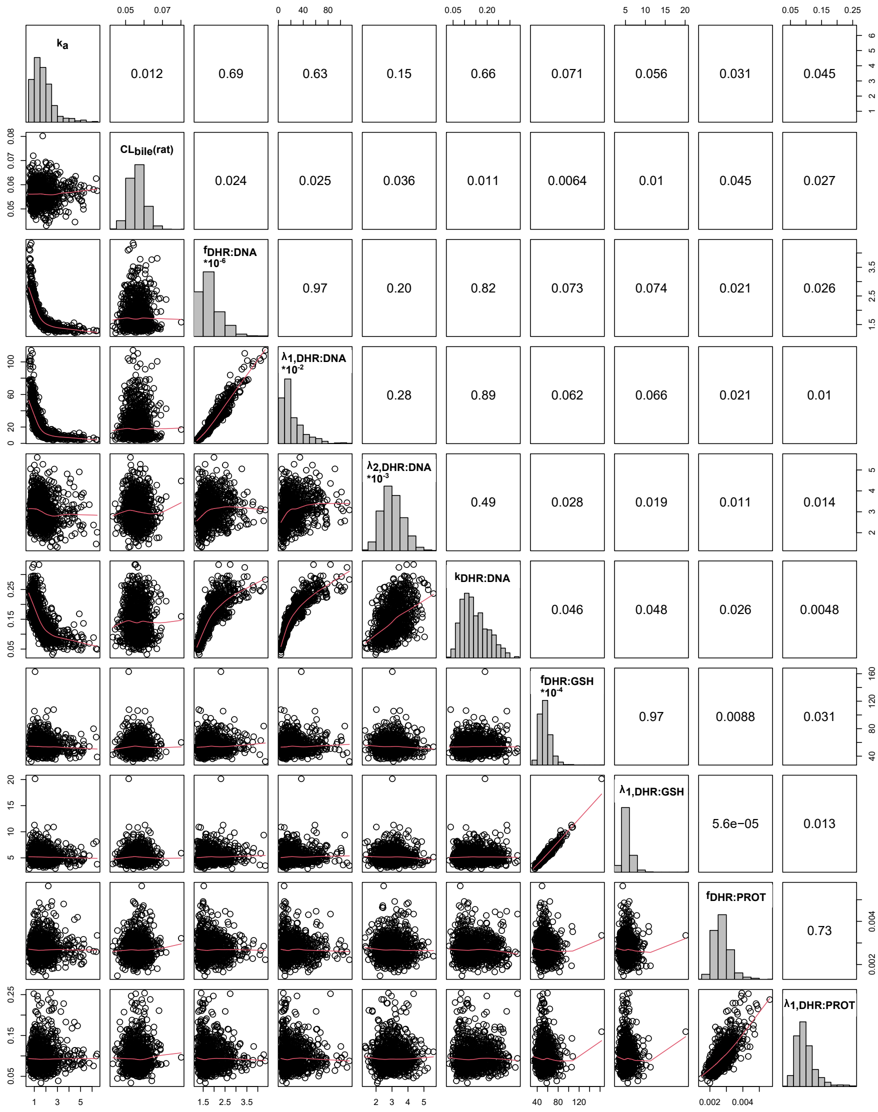

Fig. S3 Pairs plot of 1000 Monte Carlo samples of estimated parameter distributions of the PBTK model of retrorsine. **Diagonal**: histograms of the marginal posterior distribution of the parameters. **Lower triangle**: pairwise correlation plots with Loess smooth (red line). **Upper triangle**: Pearson correlation coefficients

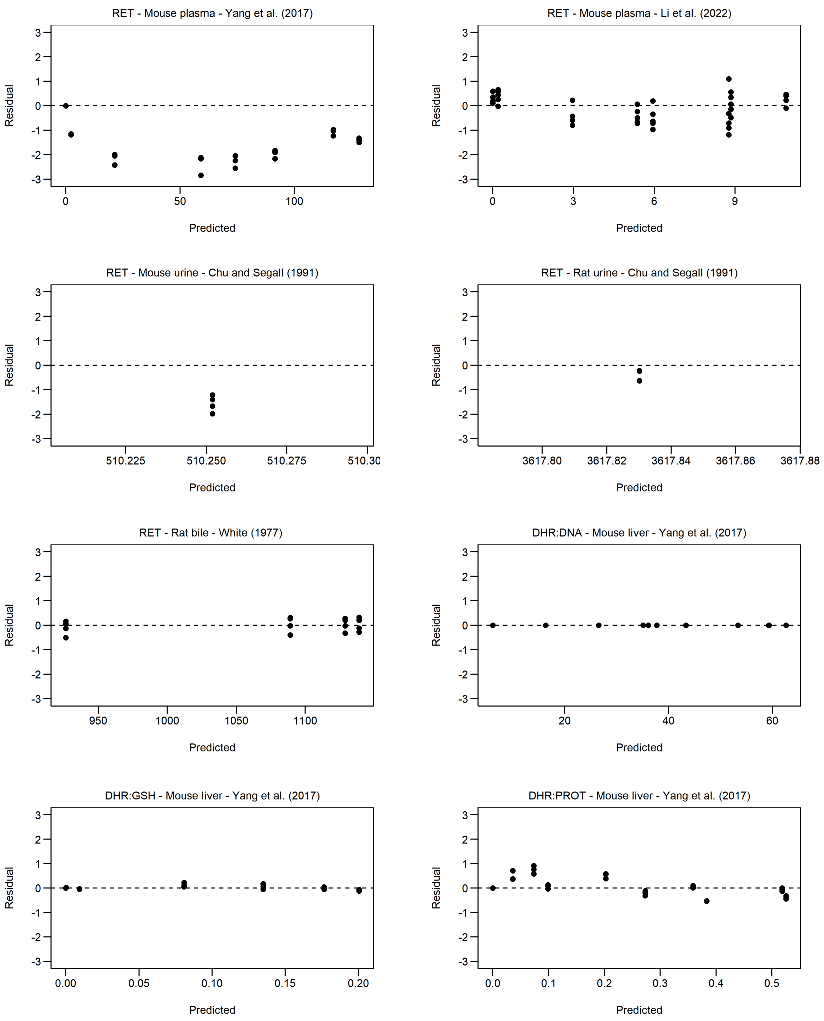

Fig. S4 Residual plots (Predicted vs. Residual) of the PBTK model fit using the training data

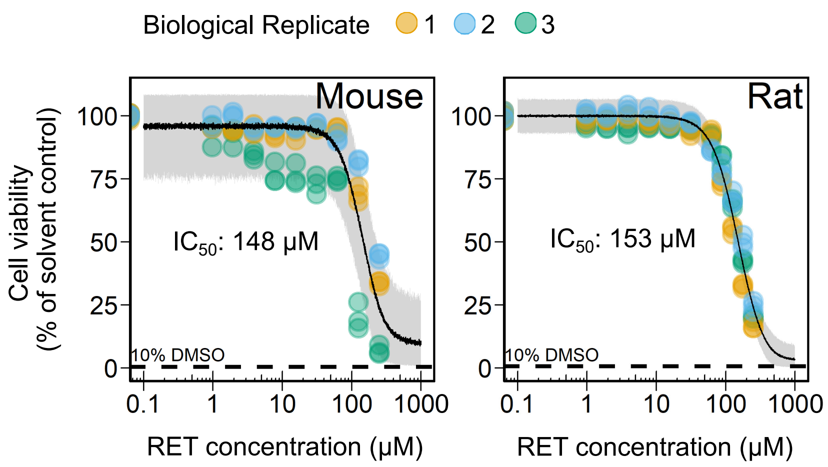

Fig. S5 Cell viability of primary mouse and rat hepatocytes in dependence of retrorsine (RET) concentration described by the sigmoidal inhibition model (Eq. S36). Cells were treated with RET concentrations ranging from 1 to 250 μM and cell viability (% of solvent control; 1.25% acetonitrile) was analyzed with the CellTiter-Blue® fluorescence assay. The dashed line represents the cell viability after treatment with 10% DMSO (positive control; dimethyl sulfoxide). Plots show observations of n=3 biological replicates, each assessed with n=3 technical replicates per concentration

Mouse

Fitted Models

model	converged	loglik	npar	AIC
full model	yes	-31.15	11	84.30
null model	yes	-76.88	2	157.76
Expon. m3-	yes	-32.48	4	72.96
Expon. m5-	yes	-31.76	5	73.52
Hill m3-	yes	-32.47	4	72.94
Hill m5-	yes	-31.84	5	73.68
Inv.Expon. m3-	yes	-32.22	4	72.44
Inv.Expon. m5-	yes	-32.69	5	75.38
LN m3-	yes	-32.33	4	72.66
LN m5-	yes	-32.18	5	74.36

Weights for Model Averaging

EXP	HILL	INVEXP	LOGN
0.22	0.23	0.29	0.26

Final BMD Values

endpoint	subgroup	BMDL	BMDU
Liver.integrity....	all	24.1	88.5

Rat

Fitted Models

model	converged	loglik	npar	AIC
full model	yes	119.41	13	-212.82
null model	yes	-73.60	2	151.20
Expon. m3-	yes	118.53	4	-229.06
Expon. m5-	yes	118.66	5	-227.32
Hill m3-	yes	118.57	4	-229.14
Hill m5-	yes	118.66	5	-227.32
Inv.Expon. m3-	yes	112.32	4	-216.64
Inv.Expon. m5-	yes	70.11	5	-130.22
LN m3-	yes	118.29	4	-228.58
LN m5-	yes	113.89	5	-217.78

Weights for Model Averaging

EXP	HILL	INVEXP	LOGN
0.35	0.37	0	0.28

Final BMD Values

endpoint	subgroup	BMDL	BMDU
Liver.integrity....	all	79.9	104

Fig. S6 Tabular reports of benchmark dose analysis results for mouse and rat generated by the web application `https://r4eu.efsa.europa.eu/app/bmd`

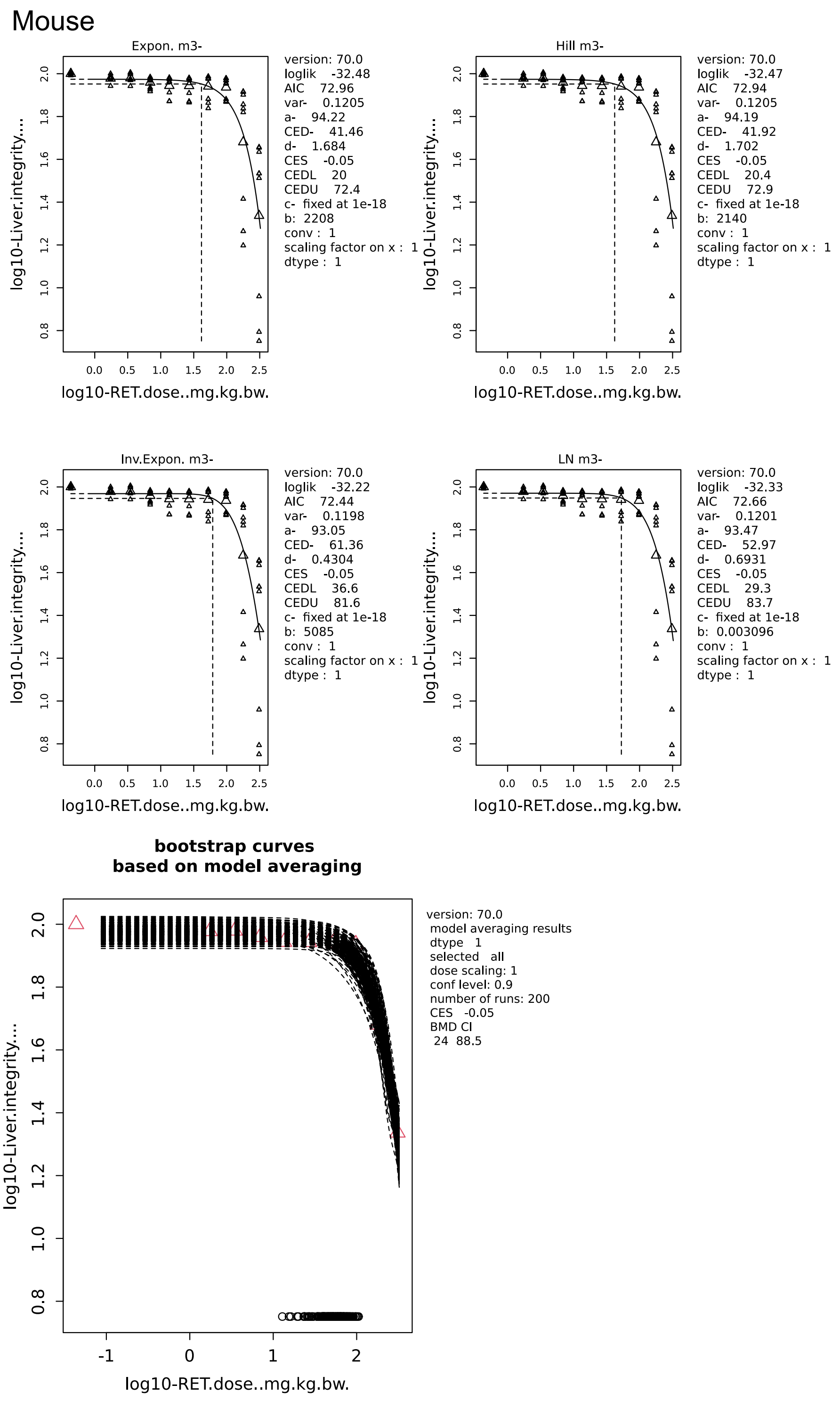

Fig. S7 Plots of benchmark dose analysis results for the mouse generated by the web application `https://r4eu.efsa.europa.eu/app/bmd`. Plots on the top show the four models used for model averaging. The plot at the bottom shows results from 200 bootstrap runs based on model averaging

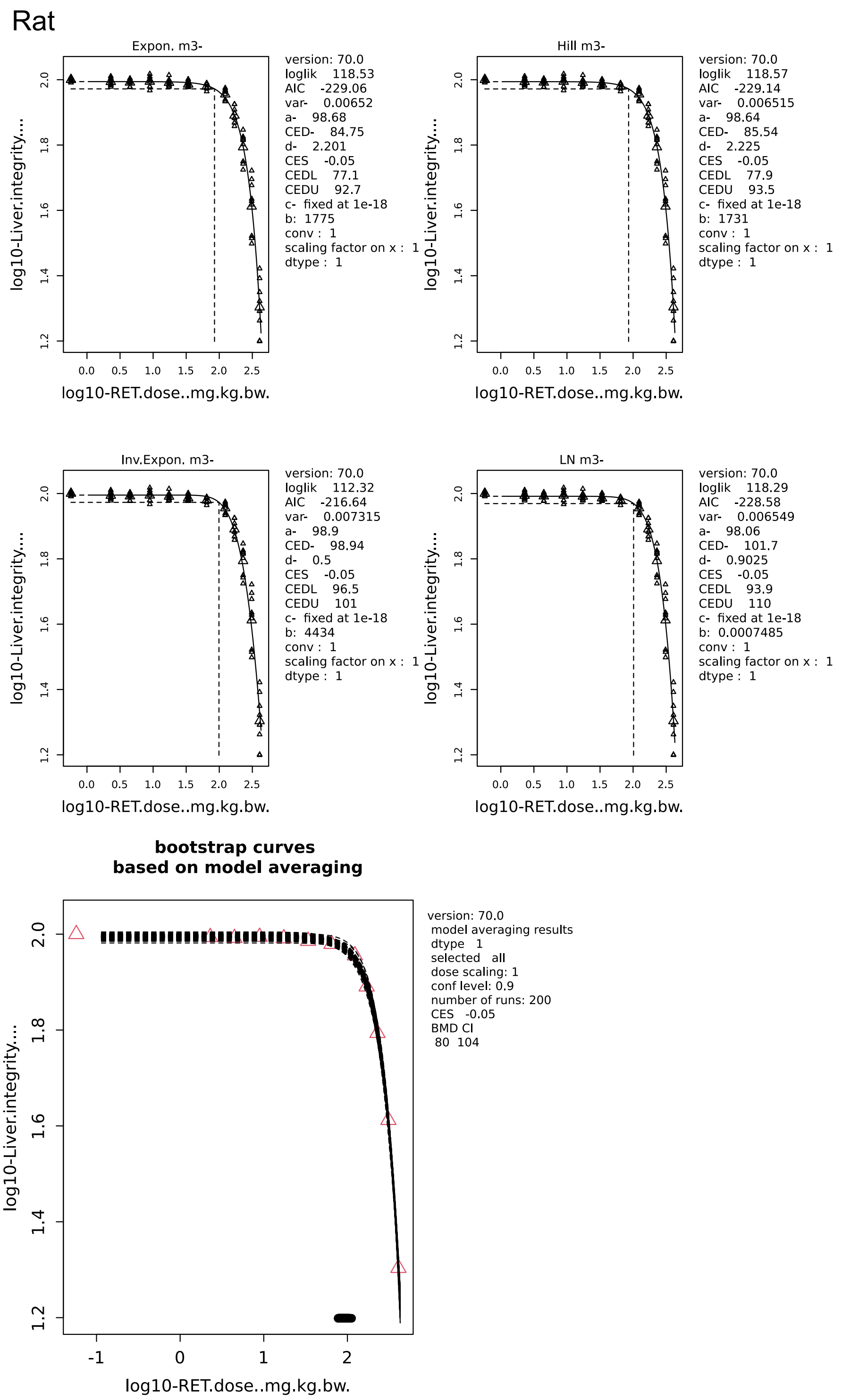

Fig. S8 Plots of benchmark dose analysis results for the rat generated by the web application `https://r4eu.efsa.europa.eu/app/bmd`. Plots on the top show the four models used for model averaging. The plot at the bottom shows results from 200 bootstrap runs based on model averaging

References

ARChem (2019) SPARC. http://www.archemcalc.com/sparc.html

Austin RP, Barton P, Mohmed S, Riley RJ (2005) The binding of drugs to hepatocytes and its relationship to physicochemical properties. Drug Metab Dispos 33(3):419–425, https://doi.org/10.1124/dmd.104.002436

Baskurt OK, Hardeman MR, Rampling MW, Meiselman HJ (2007) Handbook of Hemorheology and Hemodynamics - Volume 69 Biomedical and Health Research, 1st edn. IOS Press, Amsterdam

Benjamin A, Gallacher DJ, Greiter-Wilke A, Guillon JM, Kasai C, Ledieu D, Levesque P, Prelle K, Ratcliffe S, Sannajust F, Valentin JP (2015) Renal studies in safety pharmacology and toxicology: A survey conducted in the top 15 pharmaceutical companies. Journal of Pharmacological and Toxicological Methods 75:101–110, https://doi.org/10.1016/j.vascn.2015.01.004

Brown RP, Delp MD, Lindstedt SL, Rhomberg LR, Beliles RP (1997) Physiological parameter values for physiologically based pharmacokinetic models. Toxicol Ind Health 13(4):407–484, https://doi.org/10.1177/074823379701300401

Chu PS, Segall HJ (1991) Species difference in the urinary excretion of isatinecic acid from the pyrrolizidine alkaloid retrorsine. Comp Biochem Physiol C, Comp Pharmacol Toxicol 100(3):683–686, https://doi.org/10.1016/0742-8413(91)90061-W

Dai J, Zhang F, Zheng J (2010) Retrorsine, but not monocrotaline, is a mechanism-based inactivator of P450 3A4. Chemico-Biological Interactions 183(1):49–56, http://doi.org/10.1016/j.cbi.2009.10.001

Diehl KH, Hull R, Morton D, Pfister R, Rabemampianina Y, Smith D, Vidal JM, van de Vorstenbosch C, European Federation of Pharmaceutical Industries Association and European Centre for the Validation of Alternative Methods (2001) A good practice guide to the administration of substances and removal of blood, including routes and volumes. J Appl Toxicol 21(1):15–23, https://doi.org/10.1002/jat.727

El-Masri HA, Portier CJ (1998) Physiologically based pharmacokinetics model of primidone and its metabolites phenobarbital and phenylethylmalonamide in humans, rats, and mice. Drug Metab Dispos 26(6):585–594, https://dmd.aspetjournals.org/content/26/6/585.long

Geburek I, Preiss-Weigert A, Lahrssen-Wiederholt M, Schrenk D, These A (2020) In vitro metabolism of pyrrolizidine alkaloids – Metabolic degradation and GSH conjugate formation of different structure types. Food and Chemical Toxicology 135:110868, https://doi.org/10.1016/j.fct.2019.110868

Godoy P, Hewitt NJ, Albrecht U, Andersen ME, Ansari N, Bhattacharya S, Bode JG, Bolleyn J, Borner C, Böttger J, Braeuning A, Budinsky RA, Burkhardt B, Cameron NR, Camussi G, Cho CS, Choi YJ, Craig Rowlands J, Dahmen U, Damm G, Dirsch O, Donato MT, Dong J, Dooley S, Drasdo D, Eakins R, Ferreira KS, Fonsato V, Fraczek J, Gebhardt R, Gibson A, Glanemann M, Goldring CEP, Gómez-Lechón MJ, Groothuis GMM, Gustavsson L, Guyot C, Hallifax D, Hammad S, Hayward A, Häussinger D, Hellerbrand C, Hewitt P, Hoehme S, Holzhütter HG, Houston JB, Hrach J, Ito K, Jaeschke H, Keitel V, Kelm JM, Kevin Park B, Kordes C, Kullak-Ublick GA, LeCluyse EL, Lu P, Luebke-Wheeler J, Lutz A, Maltman DJ, Matz-Soja M, McMullen P, Merfort I, Messner S, Meyer C, Mwinyi J, Naisbitt DJ, Nussler AK, Olinga P, Pampaloni F, Pi J, Pluta L, Przyborski SA, Ramachandran A, Rogiers V, Rowe C, Schelcher C, Schmich K, Schwarz M, Singh B, Stelzer EHK, Stieger B, Stöber R, Sugiyama Y, Tetta C, Thasler WE, Vanhaecke T, Vinken M, Weiss TS, Widera A, Woods CG, Xu JJ, Yarborough KM, Hengstler JG

(2013) Recent advances in 2D and 3D in vitro systems using primary hepatocytes, alternative hepatocyte sources and non-parenchymal liver cells and their use in investigating mechanisms of hepatotoxicity, cell signaling and ADME. Arch Toxicol 87(8):1315–1530, https://doi.org/10.1007/s00204-013-1078-5

Gu X, Albrecht W, Edlund K, Kappenberg F, Rahnenführer J, Leist M, Moritz W, Godoy P, Cadenas C, Marchan R, Brecklinghaus T, Pardo LT, Castell JV, Gardner I, Han B, Hengstler JG, Stoeber R (2018) Relevance of the incubation period in cytotoxicity testing with primary human hepatocytes. Arch Toxicol 92(12):3505–3515, https://doi.org/10.1007/s00204-018-2302-0

Haas M, Tänzer J, Hamscher G, Lehmann A, Hethey C, These A (2019) Bestimmung von stoffabhängigen Parametern zur toxikokinetischen Modellierung von Pyrrolizidinalkaloiden. Lebensmittelchemie 73(S1):S142–S142, https://doi.org/10.1002/lemi.201951142

Hartung N, Huisinga W (2019) A flexible and transparent MATLAB framework for empirical and mechanistic pharmacometric modelling. www.page-meeting.org/?abstract=9082

Hessel S, Gottschalk C, Schumann D, These A, Preiss-Weigert A, Lampen A (2014) Structure-activity relationship in the passage of different pyrrolizidine alkaloids through the gastrointestinal barrier: ABCB1 excretes heliotrine and echimidine. Mol Nutr Food Res 58(5):995–1004, https://doi.org/10.1002/mnfr.201300707

IUPAC (1997) Compendium of Chemical Terminology, 2nd edn. Blackwell Scientific Publications, Oxford, https://doi.org/10.1351/goldbook

Kawai R, Lemaire M, Steimer JL, Bruelisauer A, Niederberger W, Rowland M (1994) Physiologically based pharmacokinetic study on a cyclosporin derivative, SDZ IMM 125. J Pharmacokinet Biopharm 22(5):327–365, https://doi.org/10.1007/BF02353860

Li J, Zhou M, Lai X, Wang Y, Zou Y, Li K, Li W, Zheng J (2022) Toxicokinetic and bioavailability studies on retrorsine in mice, and ketoconazole-induced alteration in toxicokinetic properties. Biomed Chromatogr 36(2):e5270, https://doi.org/10.1002/bmc.5270

NCBI (2021a) Mouse Genome Assembly GRCm39 - Genome Reference Consortium. https://www.ncbi.nlm.nih.gov/assembly/GCF_000001635.27

NCBI (2021b) PubChem Compound Summary for CID 3016, Diazepam. https://pubchem.ncbi.nlm.nih.gov/compound/Diazepam

NCBI (2021c) PubChem Compound Summary for CID 5352411, Retrorsin. https://pubchem.ncbi.nlm.nih.gov/compound/Retrorsin

NCBI (2021d) PubChem Compound Summary for CID 97536, s-Hexylglutathione. https://pubchem.ncbi.nlm.nih.gov/compound/s-Hexylglutathione

Obach RS, Reed-Hagen AE (2002) Measurement of Michaelis constants for cytochrome P450-mediated biotransformation reactions using a substrate depletion approach. Drug Metab Dispos 30(7):831–837, https://doi.org/10.1124/dmd.30.7.831

Poulin P, Theil FP (2002) Prediction of pharmacokinetics prior to in vivo studies. 1. Mechanism-based prediction of volume of distribution. J Pharm Sci 91(1):129–156, https://doi.org/10.1002/jps.10005

Ring BJ, Chien JY, Adkison KK, Jones HM, Rowland M, Jones RD, Yates JWT, Ku MS, Gibson CR, He H, Vuppugalla R, Marathe P, Fischer V, Dutta S, Sinha VK, Björnsson T, Lavé T, Poulin P (2011) PhRMA CPCDC initiative on predictive models of human pharmacokinetics, part 3: Comparative assessement of prediction methods of human clearance. J Pharm Sci 100(10):4090–4110, https://doi.org/10.1002/jps.22552

Rodgers T, Rowland M (2006) Physiologically based pharmacokinetic modelling 2: Predicting the tissue distribution of acids, very weak bases, neutrals and zwitterions. J Pharm Sci

95(6):1238–1257, https://doi.org/10.1002/jps.20502

Schweinoch D (2014) Prediction of the hepatic uptake clearance using a compartmental modelling approach based on in vitro assay data. Master's thesis, University of Potsdam

Skolnik S, Lin X, Wang J, Chen XH, He T, Zhang B (2010) Towards prediction of in vivo intestinal absorption using a 96-well Caco-2 assay. J Pharm Sci 99(7):3246–3265, https://doi.org/10.1002/jps.22080

Turner D, Rotami-Hodjegan A, Tucker G, Yeo K (2007) Prediction of nonspecific hepatic microsomal binding from readily available physicochemical properties. Drug Metabolism Reviews 38(S1)(162), https://www.certara.com/poster/prediction-of-non-prediction-of-non-specific-hepatic-microsomal-binding-from-readily-specific-hepatic-

Verma R, Chakraborty D (2008) Alterations in DNA, RNA and protein contents in liver and kidney of mice treated with ochratoxin and their amelioration by Emblica officinalis aqueous extract. Acta Pol Pharm 65(1):3–9, https://www.ptfarm.pl/pub/File/Acta_Poloniae/2008/1/003.pdf

White IN (1977) Excretion of pyrrolic metabolites in the bile of rats given the pyrrolizidine alkaloid retrorsine or the bis-N-ethylcarbamate of synthanecine A. Chem Biol Interact 16(2):169–180, https://doi.org/10.1016/0009-2797(77)90126-0

Yang X, Li W, Sun Y, Guo X, Huang W, Peng Y, Zheng J (2017) Comparative Study of Hepatotoxicity of Pyrrolizidine Alkaloids Retrorsine and Monocrotaline. Chem Res Toxicol 30(2):532–539, https://doi.org/10.1021/acs.chemrestox.6b00260

Yang X, Li W, Li H, Wang X, Chen Y, Guo X, Peng Y, Zheng J (2018) A Difference in Internal Exposure Makes Newly Weaned Mice More Susceptible to the Hepatotoxicity of Retrorsine Than Adult Mice. Chem Res Toxicol 31(12):1348–1355, https://doi.org/10.1021/acs.chemrestox.8b00220

Zhao YH, Abraham MH, Le J, Hersey A, Luscombe CN, Beck G, Sherborne B, Cooper I (2003) Evaluation of rat intestinal absorption data and correlation with human intestinal absorption. European Journal of Medicinal Chemistry 38(3):233–243, DOI 10.1016/S0223-5234(03)00015-1

Zhu L, Xue J, Xia Q, Fu PP, Lin G (2017) The long persistence of pyrrolizidine alkaloid-derived DNA adducts in vivo: Kinetic study following single and multiple exposures in male ICR mice. Arch Toxicol 91(2):949–965, https://doi.org/10.1007/s00204-016-1713-z
